# How generalisable are material extrusion additive manufacturing parameter optimisation studies? A systematic review

**DOI:** 10.1016/j.heliyon.2022.e11592

**Published:** 2022-11-18

**Authors:** Mark Golab, Sam Massey, James Moultrie

**Affiliations:** Institute for Manufacturing, Department of Engineering, University of Cambridge, UK

**Keywords:** Material extrusion additive manufacturing, Fused deposition modelling, Filament behaviour, Printing parameters, Dimensional accuracy, Review

## Abstract

**Goal:**

Material extrusion additive manufacturing, is a relatively inexpensive and popular manufacturing technique that can be used to fabricate complex 3D geometries at low cost. However, parts produced by this process are often characterised by poor quality, particularly with regards to dimensional and geometrical accuracy. This review provides a comprehensive analysis of experimental studies conducted over the past 25 years that have aimed to improve these quality variables via printing parameter optimisation.

**Methods:**

An initial non systematic scoping study coupled with a subsequent scientific systematic literature review protocol to identify experimental studies on dimensional quality in material extrusion additive manufacturing was conducted. 127 individual studies are identified and analysed.

**Results:**

The authors critically analysed the relevant and salient studies (127) by evaluating which machines; materials; sample sizes; artefact designs; and most importantly what printing parameters have been used in the experimental investigations. A total of (79) machine variations were used; ABS and PLA made up (43%) and (36%) of materials investigated respectively; (84%) of studies had sample sizes of less than (40); and artefact dimensions ranged from (10–270 mm) (1–240 mm), and (3.5–220 mm) in the X, Y, and Z axes respectively. In many cases, the relationships between printing parameters (independent variables) and dimensional qualities (dependent variables) were found to be uncertain or even contradictory between studies.

**Conclusions:**

A wide range of studies have sought to optimise parameters (e.g., Nozzle gap height, print head velocity, filament volumetric velocity) to address dimensional quality issues in ME AM. However, the authors have demonstrated that a lack of agreement among studies limits the generalisability of these parameter optimisation findings. More recent studies have considered the local dimensional variance of deposited single strands. This offers greater potential to understand the underlying causes of component defects and inaccuracy.

## Highlights

• Studies investigating ME AM part quality show no consistency in methods and results.
• Prior work is not widely generalisable; each study has a specific experimental set-up.
• Nozzle-gap-height, print-head-speed & filament-volumetric-speed are key parameters.
• There is a lack of agreement about how to optimise key parameters.
• Single strand behaviour and underlying mechanisms of part quality need further study.

## 1 Introduction

Material Extrusion Additive Manufacturing (ME AM) also known as fused deposition modelling (FDM), fused filament fabrication (FFF), and fused layer modelling (FLM) is one of the most popular AM techniques, especially amongst non industrial users [1]. ME AM is regarded as inexpensive in comparison to alternative AM techniques, whereby printers can cost as little as USD150. Due to the proliferation of this low cost technology, it is estimated, that by 2027, the ME AM market of “non hobbyist and consumer” users will generate USD2.2B in printer and hardware sales [2].

**Figure 1:**
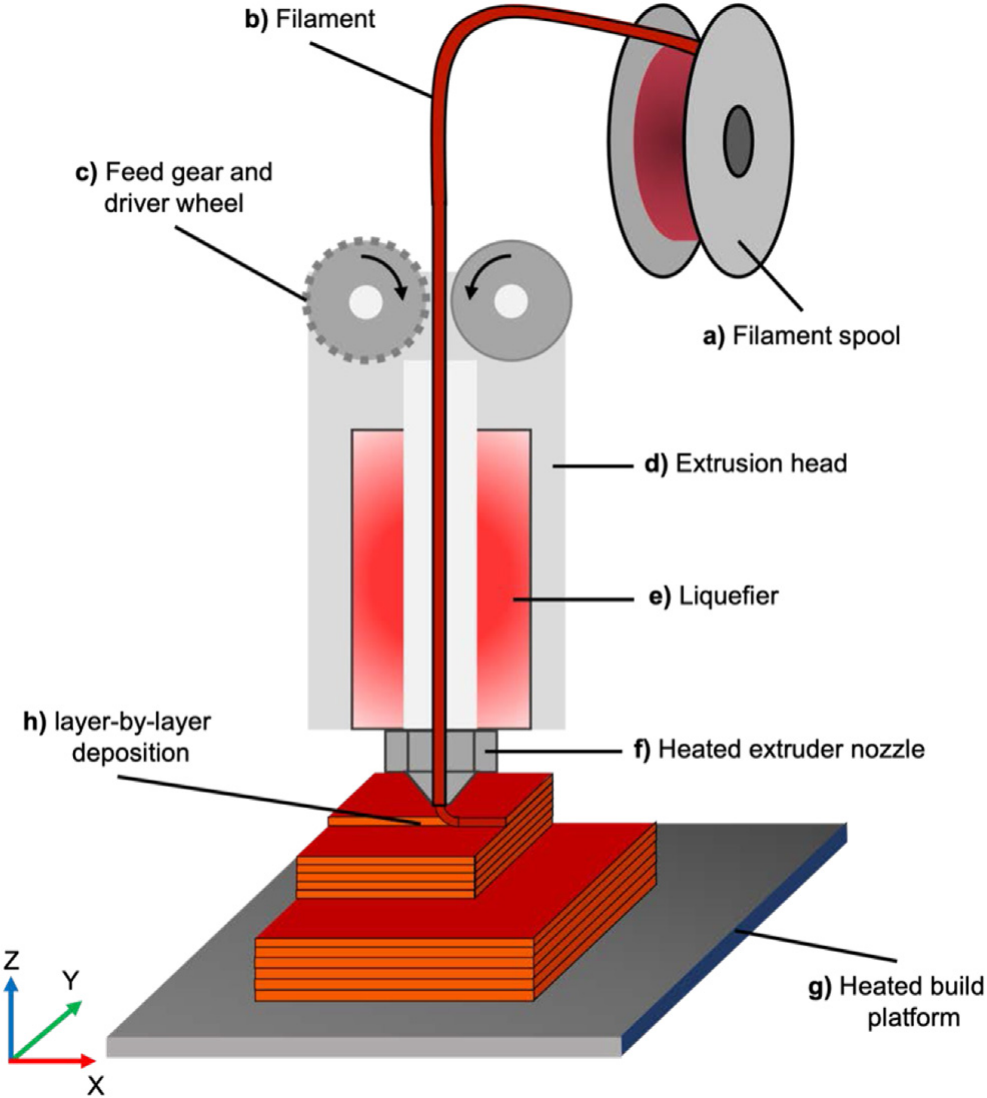
ME AM process schematic illustration. The components are labelled as: a) filament spool, which holds the filament. b) filament, the material in its solid date. c) Feed gear and drive wheel, filament is hauled off into the liquefier. d) Extrusion head encompasses all the components. e) Liquefier heats up the filament to its molten state. f) Heated extruder nozzle, filament is extruded. g) Heated build platform, filament is selectively deposited onto. h) layer-by-layer deposition, object is manufactured in layers.

Figure 1 depicts an illustrative schematic of the ME AM extrusion process and its components.

ME AM is seen as an attractive alternative to many conventional manufacturing technologies because of a number of intrinsic and well documented advantages: convenience, versatility, multi material parts, and reduced time to market. Moreover, it is a comparatively low cost way to produce parts in small volumes and for prototypes due to cheaper hardware and raw materials [3, 4]. However, there are also some significant limitations that prevent ME AM being adopted as a primary production process. The process is also considered to have some mechanical performance limitations, especially anisotropic strength, and stiffness with poor performance in the build direction. ME AM is significantly slower than that of conventional mass manufacturing techniques such as injection moulding [5].

One of the most significant limitations that inhibits uptake for production quality components relates to the achievement of dimensional quality (DQ), including poor dimensional and geometrical accuracy, Z axis resolution, surface finish and low levels of process precision [6, 7]. Poor DQ is one of the most commonly referenced limitations and substantial efforts are being undertaken in addressing it [8]. ME AM parts need to meet the requirements that they have been engineered for, if they don't fit together or exhibit defects i.e., poor DQ, they will not be functionally purposeful. ME AM parts should match the nominal dimensions set prior to manufacturing. Attempts to improve component build time by optimising parameters such as increasing the layer height or increasing print speed, often have a detrimental impact on dimensional accuracy (DA).

Recognising the importance of improved DQ as ME AM continues to develop; this review article focuses exclusively on examining literature relating to DQ. Of the DQ limitations, poor DA is especially regarded as a significant boundary for full acceptance [9, 10]. For example, one study reported that ME AM fabrication has a failure rate of 41.1%, whereby ∼25% of total failures were due to poor DQ as a consequence of processing difficulties [11]. It is widely acknowledged throughout existing literature that dimensional quality errors are significantly influenced via printing parameter selection [12, 13, 14].

Whilst it is recognised that ME AM suffers from poor DQ, there are multiple methods of addressing this issue. It should first be noted that there is a distinction to be made between studies which solely characterise the current performance of the process ‘error analysis’ and those which aim to make improvements in some way (‘error improvement’) [15]. Amongst error improvement studies, a further categorisation can be made; ‘error avoidance’, ‘error compensation’ [16] and post processing. This review focuses specifically on parameter optimisation studies, a major area of research within error avoidance.

Currently, although many studies have been undertaken there is no clear consensus on which printing parameters have the greatest effect on DQ, what the optimal values of these are and what the magnitude and nature of the remaining errors are when optimisations are made. This review aims to benefit a wide audience. Commercial interests, researchers and hobbyists can all use its findings to gain insights in the state of the art research being undertaken in improving the ME AM process. We also hope that this review is of use to academics in this domain to help focus and steer future work in a beneficial direction. Thus, in this review:

• The authors demonstrate a comprehensive analysis of the state of the art experimental studies that have made efforts in elucidating poor DQ as a function of printing parameter modulation.
• The authors provide a detailed synthesis on how DQ is significantly influenced by an increase and/or decrease in certain printing parameter values.
• The authors critically examine the landmark studies that are exploring novel approaches to understanding and enhancing DQ and precision of ME AM produced parts.
• The authors discuss the complexities in optimising printing parameters for improving the DQ of printed parts and the scope for future work.

## 2 Literature search methods

### 2.1 Literature review search strategy

In order to critically analyse extant literature which examines the DQ of printed parts, a rigorous systematic literature review was completed. To ensure that all relevant studies were identified, two review approaches were undertaken. Initially, a non systematic scoping study also known as a mapping review was used. A scoping study is defined as a review of a body of literature that can be of particular use when the topic has not yet been extensively reviewed [17]. This was performed to assess the subject area in general and to determine key areas requiring further investigation. This provided an initial foundation for this review. This initial non systematic scoping study identified which printing parameters have previously been investigated and shown to influence the DQ, and therefore informed keywords and synonyms for the second review approach.

### 2.2 Systematic literature review protocol

Systematic reviews aim to identify, evaluate, and summarize the findings of all relevant individual studies within a specific subject area, thereby making the available evidence more accessible to the wider community in order to make informed future decisions [18]. A rigorous systematic literature review protocol was conducted using a targeted search strategy by employing the Scopus search database. Scopus has the largest abstract and citation database of peer-reviewed literature, which includes scientific journals, books, and conference proceedings. Thus, due to this extensive database, Scopus was chosen as the search engine for this systematic literature review protocol. This review coupled search terms (“Material Extrusion Additive Manufacturing”, “ME AM”, “Fused Deposition Modelling”, “FDM”, “Fused Filament Fabrication”, “FFF”, “Fused Layer Modelling”, “FLM”, “3D Printing”, “Rapid Prototyping”) with key words (“Dimensional Accuracy”, “Geometrical Accuracy”, “Part Quality”, “Dimensional Variation”, “Dimensional Tolerance”) through the use of the Boolean operator (AND); e.g. “Rapid Prototyping” AND “Part Quality”. The search terms defined the AM technology and its termed synonyms, whereas the key words specified the area of interest. Initially, each new search term was input excluding key words, e.g., “ME AM”, as this returned a broader set of results. Subsequently, the key words were added systematically. The resultant documents were identified by searching for the input search term in article titles, abstracts, and keywords specifically. In order to filter the results, and identify the salient documents, the search strategy explicitly targeted articles, conference papers, and reviews. The results were subsequently reviewed by reading the documents in full. During the filtering down process, 4837 studies were rejected. Studies were rejected for being non experimental, explicitly comparing desktop machines, comparing materials, investigating mechanical properties of parts, examining surface roughness and not DA, and validating numerical models. Furthermore, if a study had already been identified in a previous search, this duplication was also rejected and not included again in the final sample.

### 2.3 Literature review methodology and results

**Figure 2:**
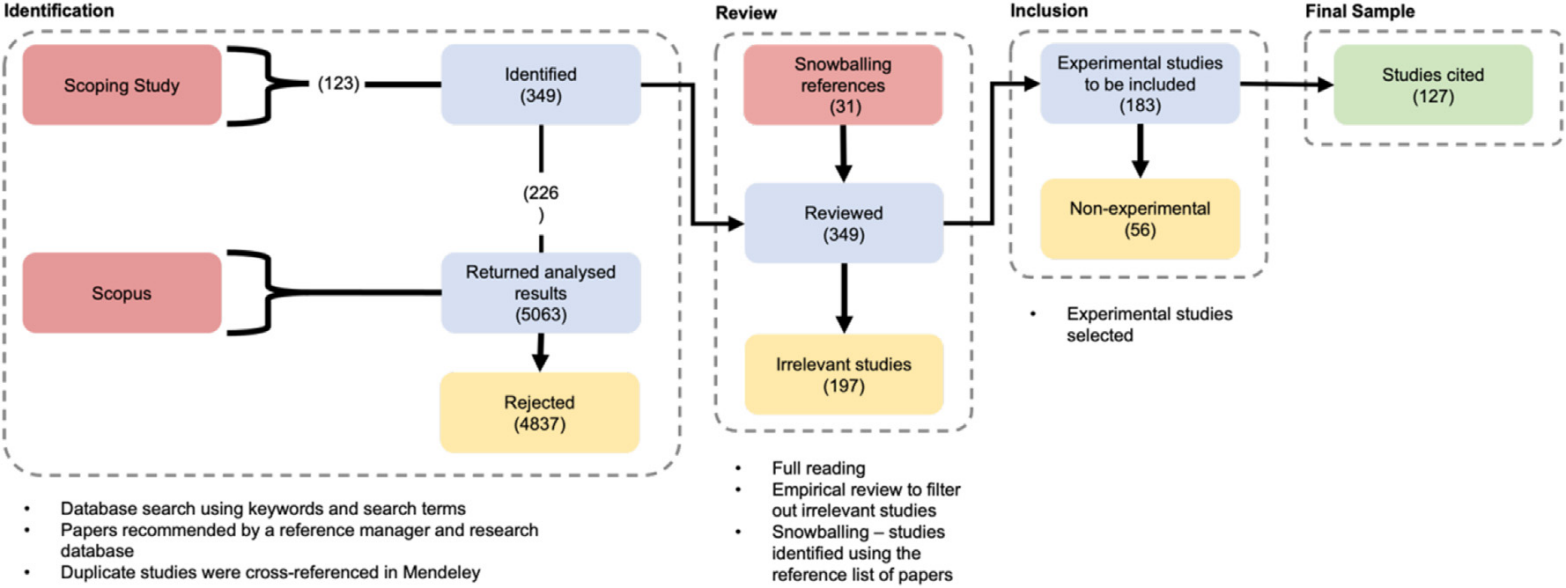
Systematic literature review methodology, adapted from the PRISMA principles [19, 20].

For the initial scoping study, 123 studies were identified. Subsequently, 5063 documents were identified by employing the Scopus review protocol. Out of the 5063 studies, 226 were determined to be of relevance. A combined total of 349 studies were therefore identified as shown in the review methodology in Figure 2. The methodology in Figure 2, illustrates the procedure used to identify the studies that were of importance and would be critically analysed in this review article.

During the review phase, additional studies were discovered using the reference lists from the reviewed studies, in a process referred to as ‘snowballing’. This identified 31 further studies which were subsequently included in the review phase. 197 studies were discarded upon further analysis as either irrelevant (i.e., studies that were not explicitly investigating process optimisation) or for being descriptive or non experimental. For example, a study which investigated the relative DQ performance of different desktop printers and materials without investigating a range of printing parameters would not be considered for inclusion. A total of 127 experimental articles were ultimately deemed directly relevant and were therefore critically analysed for the purpose of this review article.

### 2.4 Study characterisation

Each of the 127 studies that were identified in this systematic literature review were analysed to investigate what approaches had been used to characterise DQ. In order to analyse the experimental methods used in determining the relationship between the printing parameters and DQ metrics, each study was analysed, and the most important aspects were formatted as shown in Table 1. For each study the: ME AM machine type, slicer, materials used, optimisation method, independent variables, sample size, test artefact, dependent variables, and relationship were recorded. A comparison between the recorded studies was subsequently undertaken, which is detailed in section 4 onwards. The relationship column shows the interaction effects between the independent variables (printing parameters) and dependent variables (dimensional quality aspects). The full table with all 127 analysed studies can be found in Appendix A.

**Table 1:** Studies that have experimentally investigated printing parameter optimisation in ME AM.

Study	Machine	Slicer	Materials Used	Optimisation Method/Technique or Tools	Independent Variables	Characterisation Method	Sample Size	Test Artefact	Dependent Variables	Relationship between independent and dependent variables
[21]	Maxum RP Machine	N/A	ABS	Taguchi method, Analysis of Variance (ANOVA) and signal to noise ratio (SNR)	Contour width Raster width Raster angle Air gap	Mitutoyo BH303 coordinate measuring machine (CMM)	9 samples	A square benchmark model (50 × 50 mm) with surface features	Dimensional accuracy (DA)	Contour width ↑ (GF ++) Road width ↓ (DA ++) (GF ++) Raster angle ↑ (SR ++) Air gap ↑ (SR ++)
									Geometric forms (GF)	
									Surface roughness (SR)	
[22]	Vantage SE	N/A	ABS	Taguchi method, Analysis of Variance (ANOVA), signal to noise ratio, Gray Relational Analysis	Layer thickness Build orientation Raster angle Road width Air gap	Mitutoyo vernier callipers	27 samples	Cuboid 80 × 10 × 4 mm	Dimensional accuracy (DA)	Layer thickness ↑ (DA +) Build orientation ↓ (DA ++) Raster angle ↓ (DA +) Road width ↑ (DA +) Air gap ↑ (DA +)

In Table 1, the arrows (↑ ↓) indicate the direction of change of the independent variable. For example, “road width ↑” indicates an increase in road width. If there is a positive effect on the dependent variable, this is noted in column 10, which shows the magnitude of this relationship. A single plus sign (+) indicates that the change in the independent variable results in a moderate improvement in the dependent variable. A double plus sign (++) indicates that the change in the independent variable results in a large improvement in the dependent variable. For example, for study [22], an increase in layer thickness results in a moderate improvement in DA. In study [21], a reduction in road width results in a large improvement in DA.

Two studies have been highlighted in Table 1 in order to compare the similarities and differences in their approaches and findings. Both studies [21, 22] investigated the effects that “road width” has on DA, and yet their results are not in agreement with each other. Study 21 concludes that an increase in road width results in a large reduction in DA. Conversely, Study 22 concludes that an increase in road width results in a moderate improvement in DA. Thus, the findings warrant further investigation; the findings are analysed and discussed in Section 4 and onwards. The full table in Appendix A is used as a foundation to examine the various machines, materials, and sample sizes that have been used in experimentally investigating parameter optimisation.

## 3 Experimental studies investigating printing parameter optimisation

### 3.1 Citation network

**Figure 3:**
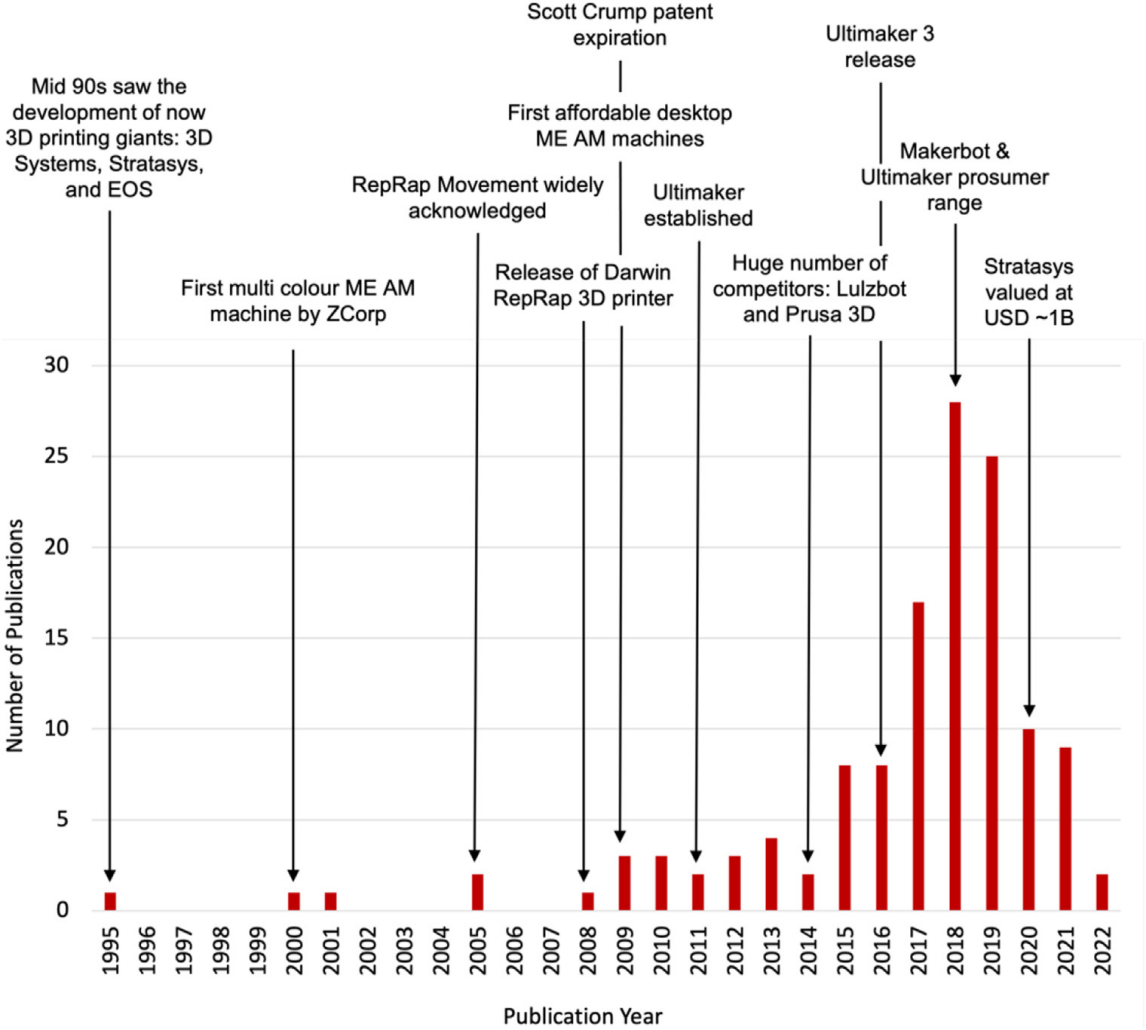
Temporal distribution of salient experimental studies identified in the literature review and timeline of notable events in the development of ME AM.

Research on ME AM's printing parameters has seen increasing attention within the last five years as shown in Figure 3. It is widely acknowledged that the print parameters used can have a significant effect on DQ [7, 23, 24]. The advent of the RepRap movement, the expiration of Scott Crump's patent, and lower cost hardware resulted in the proliferation of new machines being introduced into the consumer market. Figure 3 shows that there is a correlation between the increase in machines and experimental studies published. Essentially, as the technology has become more ubiquitous, there has been an increase in academic attention to understanding and improving its limitations.

**Figure 4:**
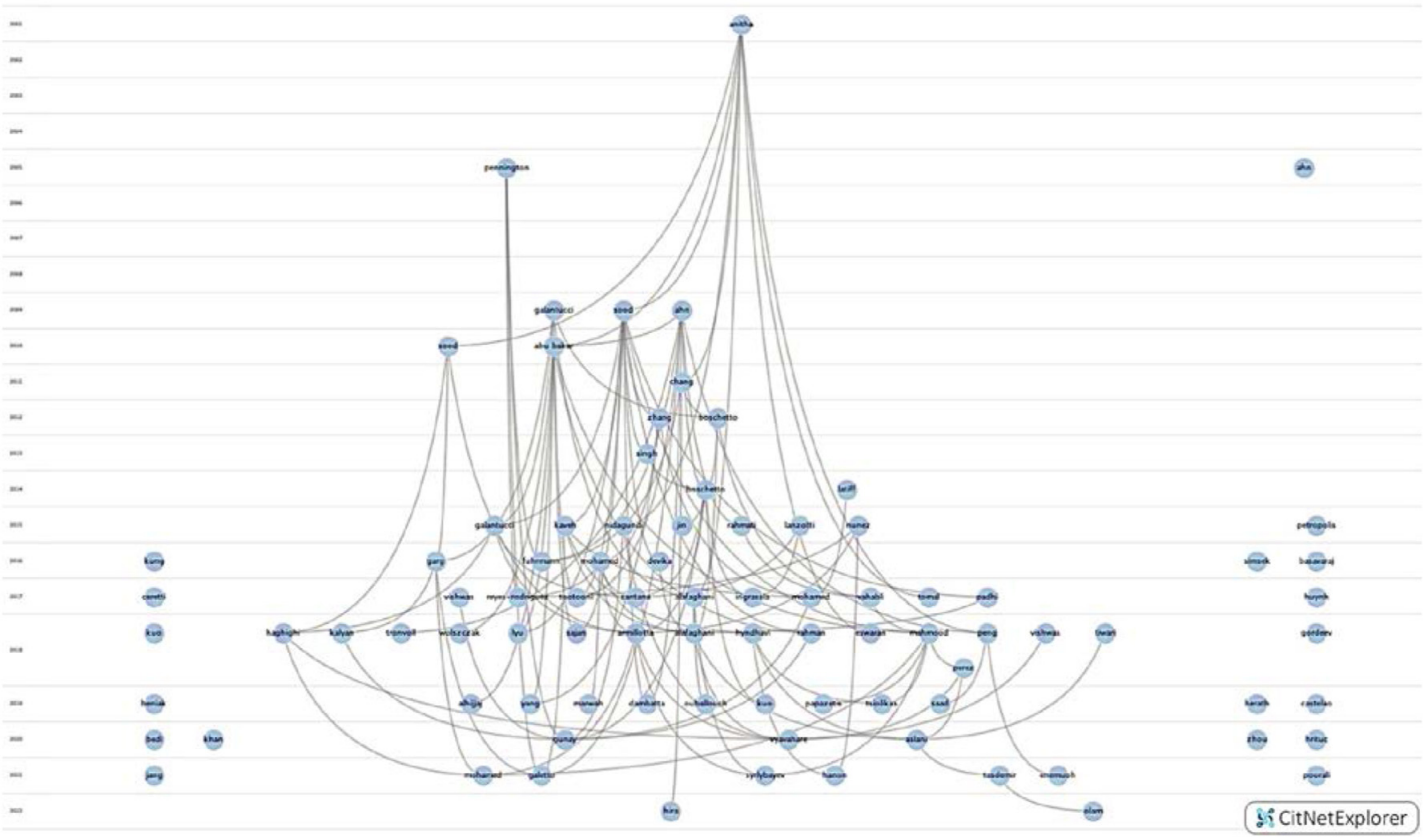
Citation network of 88 experimental studies analysed.

Of the 127 studies that experimentally investigated the DQ of printed parts, 88 were available for analysis in Web of Science's database and were subsequently imported into a CiteNetExplorer's citation network as shown in Figure 4. This software allowed the authors to visualise the citation links between the reviewed studies. The core publications in this research field as identified using the citation network are Anitha et al. [25], Sood et al. [22], Ahn et al. [26], Galantucci et al. [27], and Mahmood et al., [28].

In 2019, 12 studies that investigated DQ were published as shown in Figure 4. The 12 studies had directly cited 16 out of the 61 studies that had been published previously. This limited direct citation suggests a lack of connectivity between the studies and that each independent study is only referencing a sub set of comparable extant work. This supports the need for a thorough literature review of this type, to provide a comprehensive repository of relevant work for academics in this domain.

The empirical results demonstrate that older publications have received a greater number of citations than more recent publications. Furthermore, there is shown to be a significant increase in the number of publications in recent years. A systematic review at this stage is therefore well placed to capture the most recent efforts in this field.

### 3.2 Experimental sample size

**Figure 5:**
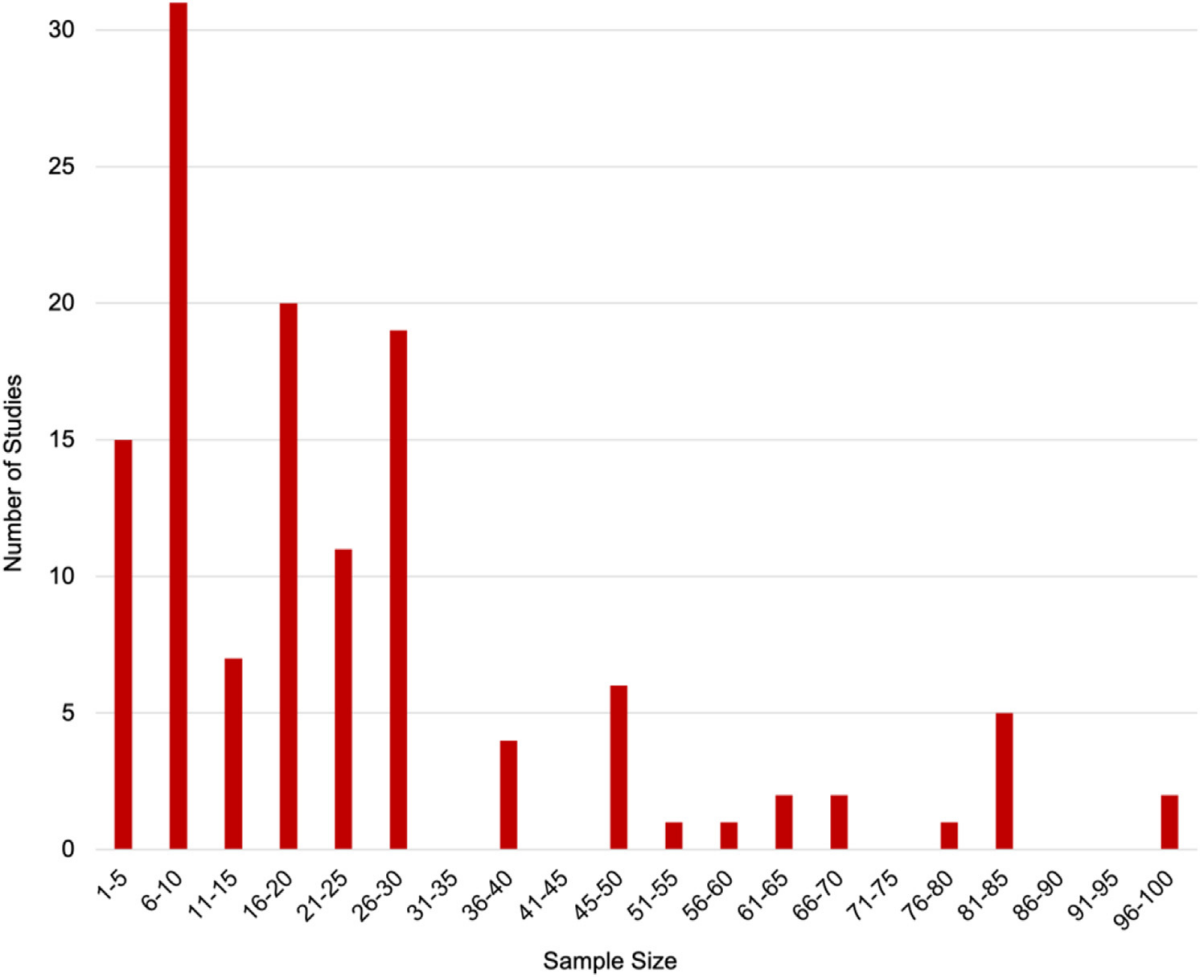
Typical printed artefact sample size used by reviewed experimental studies.

In each study, a particular sample size of experimental components is chosen. A larger sample size typically generates more data, which can help to increase the reliability of the results. However, greater sample sizes naturally require more time and effort to analyse. Figure 5 shows the sample sizes used in the 127 analysed studies. It can be observed that the majority of the studies used a sample size of between 1 and 30, whereas few studies had sample sizes greater than 36.

With multiple variables each having several levels, the Taguchi method is often employed in order to minimise the experimental complexity. This uses an orthogonal array to alter the variable levels with the minimal number of experimental tests. With a similar number of variables and levels across studies, this tends to lead to the convergence in the number of samples at around 16 to 30 for experimental approaches such as this, as shown in Figure 5.

The majority of optimisation studies perform a number of statistical techniques to establish the effects of changing parameter values. Notably, a p value is typically calculated for each variable or combination of variables. The commonly accepted value of 0.05 is used through the studies, which denotes a statistically significant likelihood of the parameter values having the measured effect on the output variable. Higher sample sizes may help to capture smaller effects, but those observed with a p value of less than 0.05 in the studies conducted can be considered statistically significant with the sample sizes used.

In some isolated studies, either a very small or a very large number of experiments have been conducted. One study with a high number of samples is Devicharan and Garg [29]. This study printed 100 identical samples of an ABS cube; the characterisation results recommended the improvements needed in the ME AM process. Alternatively, Mašović et al. [30], produced only 3 separate gear components; each gear was either printed with a different material or in a different printing direction, thus, each gear was unique. The study concluded that the printing parameters have an effect on the DA of the gears. In cases such as this where there is only a small sample size and limited statistical analysis, confidence in the results is questionable. We would strongly argue in favour of larger sample sizes for future studies in order to yield greater reliability and efficacy in the results.

### 3.3 Artefact geometry and size

In order to perform experimental work, each study produced an artefact for characterisation. These artefacts differed in both geometry and scale. The maxima, mean, and minima absolute dimensions of the parts produced across the studies are subsequently stated. In the X axis, the maxima and minima absolute part dimensions ranged from 270 mm to 10 mm respectively. In the Y axis, the maxima and minima absolute part dimensions ranged from 240 mm to 1 mm respectively. In the Z axis, the maxima and minima absolute part dimensions ranged from 220 mm to 3.5 mm respectively. The upper values here correspond to the largest build dimensions available on typical desktop ME AM machines. The mean absolute part dimensions in the X, Y, and Z axes are 54 mm, 31 mm, and 23 mm respectively.

It is interesting to note that on the whole, each study has developed its own bespoke test artefact in order to evaluate the impact of the variables being investigated in that particular study. Overall, there is no consistency in the artefacts used, with huge variation in both size and shape. Some of the most widely used artefacts used are the dog bone [31, 32, 33, 34] and the cube [10, 35, 36]. This lack of consistency has a significant impact on the comparability of results across the different studies. For example, Marwah et al. [37], used a rectangular benchmark part comprised of various features on the top surface, whereas Herath et al. [38], characterised a complex part with extruded and spherical geometries. Although this area has not been fully explored, some studies have suggested that dimensional accuracy and precision can depend on component geometry. For example [39], Bakar et al. demonstrated that cylindrical features give the largest dimensional error and suggested that this was as a result of machine design limitations. Furthermore, many studies use contrasting materials, machines, characterisation techniques and most importantly, printing parameters adding further potential for variability.

Several studies conclude that there would be benefit in some standardisation in the geometry and size of the test artefact (e.g. [40]) but has yet to achieve widespread adoption. However, if a standard base artefact with consistent dimensions and geometries were used in all the experimental studies, a comparison between studies is far more feasible. Without greater standardisation, it is especially difficult to perform a direct comparison between printing parameters, materials, or machines. This is arguably one of the most significant challenges for future work in order to not only compare results with confidence but also to develop meaningful meta analysis of the outputs from different researchers.

### 3.4 Machine types

**Figure 6:**
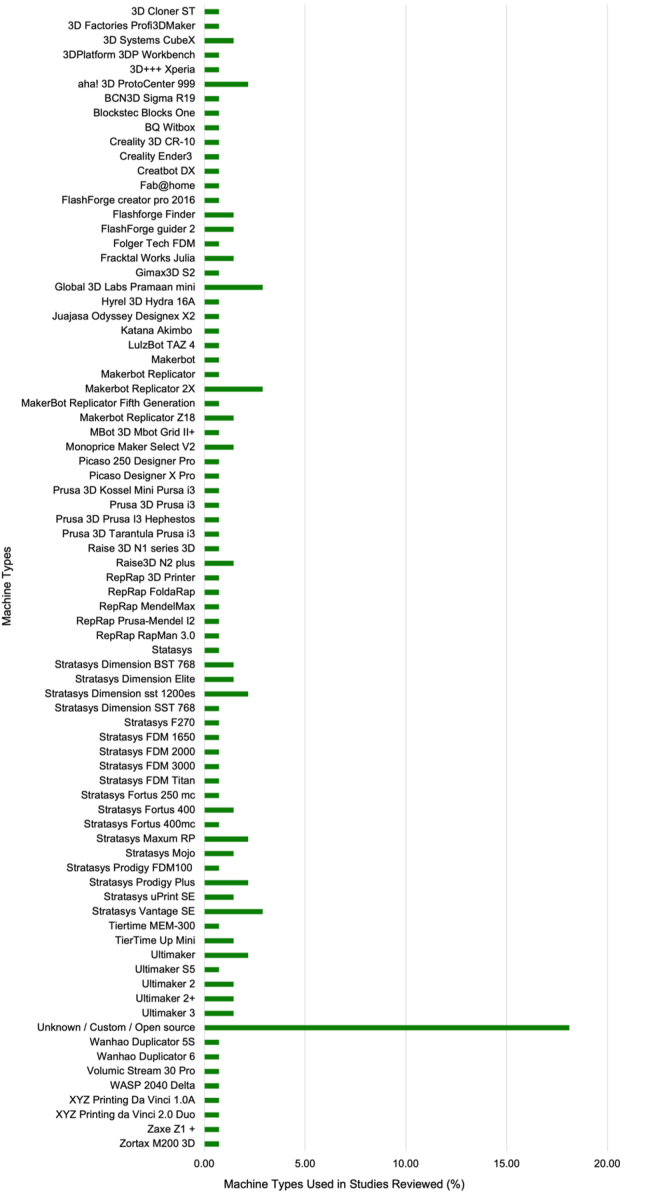
Percentage distribution of machine types used in reviewed experimental studies.

There is a plethora of ME AM machines available in the market and this is reflected in the experimental work reviewed. The range of machines used across the studies is shown in Figure 6. Of the 127 experimental studies reviewed, 76 different machine models were used to conduct experimental investigations of DQ. Some of the most popular machines used were the Stratasys Vantage SE, Global 3D Labs Pramaan mini, and Makerbot Replicator 2X. Of the 76 different machine types used, ∼97% of them were the conventional cartesian coordinate system set up rather than a delta arrangement. Although rarely documented in the studies analysed, a wide variety of Slicing software packages are available and are likely used in the experiments. The slicing software translates the 3D CAD file into instructions to drive the machine axes and each software package has a different approach to doing this. Therefore, the degree of machine software variability is likely even higher. It is important to note that each machine has its own firmware, which can influence the DA. The firmware determines the extrusion volumetric rate and the translational velocity of the extruder head, which has a nominal velocity change threshold i.e., acceleration to deceleration profile. This is commonly known as jerk [41].

It would be challenging to compare which ME AM machines have a greater influence on the DQ of produced parts without standardising other variables such as: material; artefact design; printing parameters; build orientation; and characterisation method. None of the analysed studies have yet compared machines with standardised variables. However, it is well documented that poor DQ is a function of printing parameter selection and thus to a large degree, filament behaviour rather than the inherent design of the machine [7, 23, 24]. However, poor quality of machine components has been demonstrated to have a negative effect on produced parts [42].

### 3.5 Materials

**Figure 7:**
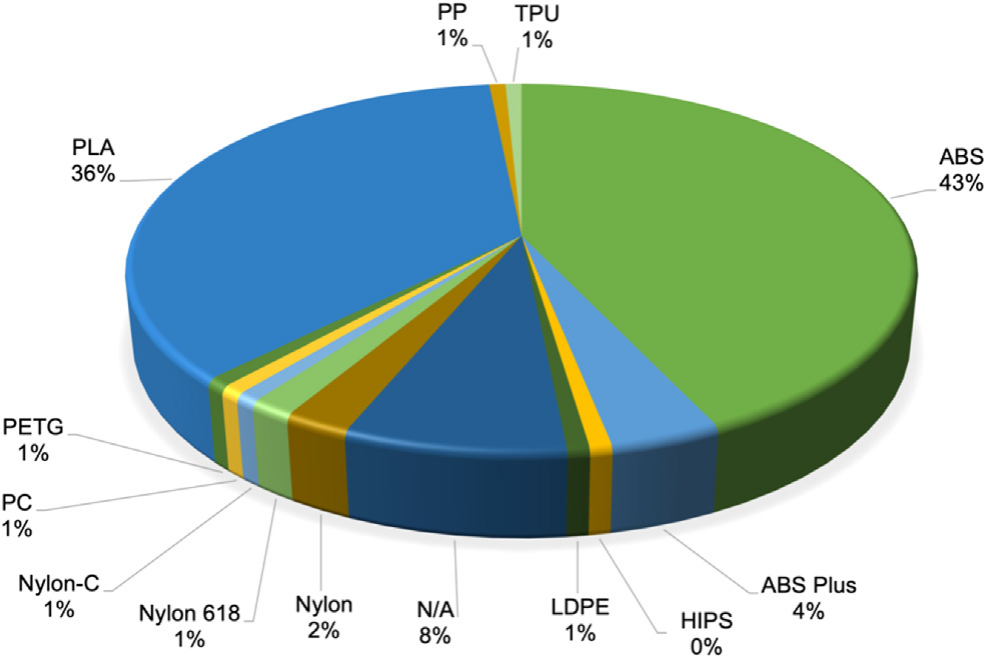
Percentage distribution of materials used in reviewed experimental studies.

A total of 12 different conventional materials were used in the 127 reviewed experimental studies. The most popular materials, as shown in Figure 7 are PLA and ABS which were used in 36% and 43% of the studies respectively. PLA and ABS are the most commercially popular ME AM materials; therefore it was to be expected that these thermoplastics would be the most widely used. Nylon was also a relatively popular material choice. PLA, ABS, and Nylon are widely used in the ME AM process due to their general ease of printing, safety, and low price point.

In addition to the conventional materials, a variety of custom and unconventional materials were used, particularly composites [43, 44, 45, 46]. The analysed studies investigated the optimum parameters for producing parts with said materials and subsequently characterised the DQ of the produced artefacts.

ME AM is heavily influenced by the choice of printing parameters, and each material behaves differently during the deposition process because of their different rheological properties. Rheological properties such as flow rate, shear rate, strength, and final material shape are considerably linked to the physical characteristics of a polymer. Some of these characteristics are density, crystallinity, and viscoelasticity. Both ABS and PLA are viscoelastic polymers, which behave in a more viscous or elastic fashion depending on the velocity in which they are deformed. However, as ABS is an amorphous polymer and PLA has a low degree of crystallinity, the melt flow behaviours of the materials differ. Particularly the temperature of the melt flows, which are influenced through viscous dissipation. Thus, resulting in both ABS and PLA having different time dependant material properties, which can consequently affect the DQ [47, 48, 49].

Furthermore, it has been demonstrated that different filament colours of the same material using constant printing parameters can also influence the DQ. This is a result of the material property differences between natural and synthetic polymers [50].

As a result, both ABS and PLA have different parameters in order to print components to the same level of DQ. Consequently, to inform the 3D printing community on how to optimise the printing parameters, the appropriate filament material must be selected according to previous studies in order to perform a direct comparison. Currently, most slicing software packages contain suggested temperatures for the selected material but do not change the nozzle path algorithm. It is therefore implicitly assumed that should these parameters be used; the geometrical performance does not differ between materials. Further work might be undertaken to elucidate if a range of materials perform in the same way for these suggested parameters.

### 3.6 Slicing software

The production of final components via the ME AM process requires the conversion of a 3D virtual model into machine code. This is then executed by the specific ME AM machine selected, of which there are many types as evidenced in the previous subsection. The production of machine code is handled by a so called ‘slicer’ or ‘slicing’ software package. This converts the outer surface mesh of the virtual model to a series of 2D slices which are then built up in order to produce the final component. Each of these slices is created by the combination of material extruded through the nozzle and the movement of that nozzle in the slice plane.

The choice of this toolpath and extrusion profile differs between slicer software packages. As a result, the final component's quality characteristics are liable to influence by the slicer software used to produce the machine code. It has been demonstrated that the slicer can have a significant effect on the accuracy of the component produced. For example [51], Baumann et al., evaluated the influence of four leading slicers on the accuracy of printed components and found large differences in the measured accuracy, particularly on overhangs and smaller features. Similarly [52], Šljivic et al., compared three slicers and again found significant differences in the end result.

**Figure 8:**
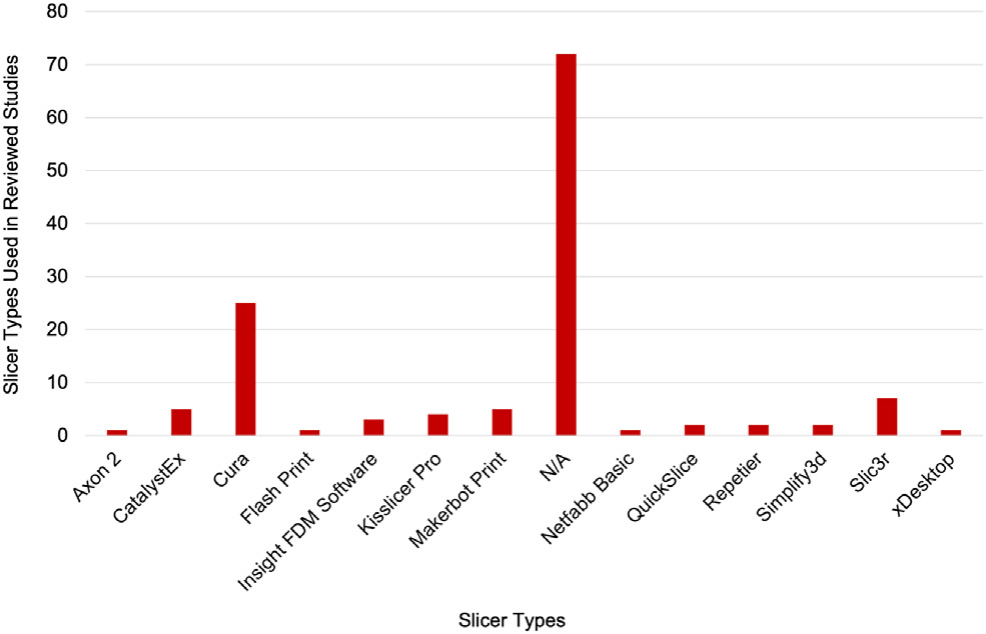
Number of slicer types used in reviewed experimental studies.

The slicer, or more specifically the toolpath and extrusion algorithms used, should therefore also be considered as process parameters which can be changed. This is analogous to the use of different component geometries and sizes, machines, and materials all of which, if not properly accounted for, will potentially influence the optimal print parameters. As with these other three factors, the studies once again demonstrate a wide variety of selections as demonstrated in Figure 8. This shows Ultimaker CURA to be the most popular, though more than half of the studies did not report the slicer used.

### 3.7 Generalisability of reviewed ME AM studies

Subsections 3.2–3.6 and Appendix A show how each study has used an entirely unique experimental set up to investigate the DQ of produced parts via parameter optimisation. Of the 127 experimental studies that were reviewed, no two studies adopted the same approach of experimental set up, i.e., machine, material, slicer, artefact, characterisation method and input variables. However, two studies [53, 54] were partially analogous with their respective experimental set ups. Both studies used identical machines, materials, and slicers. They remained different in, their respective use of artefact design, characterisation methods and independent variables. Furthermore, the findings from the two studies were somewhat contrary to one another. Anusree et al., recommended that a layer Nozzle Gap Height (NGH) of 0.15 mm improved the DQ of a printed M20 Bolt, whereas Rahman et al., concluded that a NGH of 0.2 mm improved the DQ of a standard test bar. Although the two aforementioned studies have used somewhat similar set ups, the studies cannot be directly compared, and thus the printing parameters and their interactions cannot be isolated for analysis. Differences in the experimental set ups between the 127 experimental studies demonstrate that the recommendations for improving the DQ of printed parts cannot be easily generalised and are only applicable to the specific set up used in each study. Although by its very nature, some differences between studies must be present, there is an evident lack of standardisation on other factors which are not noted. This means that direct comparisons between studies highlight the current difficulty in prescribing a general solution for improving the DQ of printed parts via parameter optimisation.

However, despite this lack of consensus, it is important to recognise that each study may present recommendations which are of significance, and therefore should be synthesised to determine correlations between printing parameters and improvements to the DQ of produced parts even if not generalisable. Generally, it is widely acknowledged that a reduced NGH improves DQ, but this is not always consistently proven, this will be discussed in greater detail in the subsequent section. It is evident from this analysis that future work would beneficially focus on using analogous experimental set ups in order to present generalisable findings.

## 4 Printing parameters and their impact

In Table 2, Table 3, and Table 4, the authors synthesise which parameters have been shown to influence DQ within the 127 studies included in this review. A full synthesis of this nature has not previously been undertaken, and the authors believe that doing so provides some new insights into the relationship between printing parameters and DQ. We also believe that is essential for other researchers in this domain in order to ensure awareness of relevant prior work and focus attention on areas for standardisation.

**Table 2:** Toolpath parameters.

Printing Parameter	Depiction	Description	Impact on dimensional accuracy identified in analysed studies from an increase (↑ top row) or decrease (↓ bottom row) in printing parameter value.	
Nozzle gap height (NGH)		Also known as layer thickness, the measured displacement from the tip of the extruder nozzle to each successive layer of deposited material. NGH can also influence the defects listed: Circularity, Cylindricity, Porosity, and Surface Roughness.	**↑**	[10, 22, 25, 32, 33, 37, 46, 60, 61, 62, 63, 64, 65, 66, 67, 68, 69, 70, 71, 72, 73, 74, 75, 76]
			↓	[12, 26, 27, 31, 34, 35, 36, 44, 45, 53, 54, 62, 77, 78, 79, 80, 81, 82, 83, 84, 85, 86, 87, 88, 89, 90, 91, 92, 93, 94, 95, 96, 97, 98, 99, 100, 101, 102, 103, 104, 105, 106, 107, 108, 109, 110, 111, 112]
Filament volumetric velocity (FVV)		Also known as feed rate, the volume of material extruded through the nozzle per unit distance travelled. FVV can also influence the defects listed: Geometrical accuracy, Porosity, and Surface Roughness.	**↑**	[38, 67, 71, 100, 105, 113, 114],
			↓	[73, 84, 103, 115]
Print head velocity (PHV)		Also known as print speed, the velocity the extruder head traversers across the build envelope during deposition and non deposition. PHV can also influence the defects listed: Circularity, Geometrical accuracy, Surface Roughness, Shrinking, and Warping.	**↑**	[25, 31, 37, 53, 54, 61, 65, 72, 73, 88, 99, 100, 109, 114, 116, 117]
			↓	[32, 34, 36, 68, 71, 76, 92, 106, 108, 112, 118]
Air Gap (AG)		The measured lateral distance between the centre points of deposited adjacent filament strands. AG can also influence the defects listed: Geometrical accuracy, Porosity, and Surface Roughness.	**↑**	[12, 21, 22, 44, 86, 110, 119, 56]
			↓	[22, 45, 46, 120]
Raster angle (RA)		The angle between deposited adjacent strands with respect to the X axis. RA can also influence the defects listed: Geometrical accuracy and Surface Roughness.	**↑**	[21, 44, 60, 88, 119, 120, 121],
			↓	[12, 22, 45, 46, 56, 66, 70, 78, 104, 110]
Road width (RW)		Also known as raster width, the width of a deposited stand, which is a function of the extruder nozzle diameter and toolpath parameters. RW can also influence the defects listed: Geometrical accuracy and Surface Roughness.	**↑**	[12, 22, 45, 53, 60, 61, 66, 83, 120, 120, 121]
			↓	[21, 25, 27, 36, 44, 46, 56, 63, 64, 110, 119, 122]
Contour width (CW)		Also known as perimeters and number of shells, the thickness of the outer wall of a printed part. CW can also influence the defects listed: Geometrical accuracy and Surface Roughness.	**↑**	[10, 21, 28, 39, 46, 85, 121, 123]
			↓	[10, 36, 38, 44, 65, 77, 87]
Contour print head velocity (CPHV)		Similar to print head velocity, this sets the velocity of the extruder head whilst depositing the contours of the part. CPHV can also influence the defects listed: Geometrical accuracy, Surface Roughness, and Warping.	**↑**	[105]
			↓	[67, 113]
Infill print head velocity (IPHV)		Similar to contour and print head velocity, this sets the velocity of the extruder head whilst depositing the infill layers of the part. IPHV can also influence the defects listed: Geometrical accuracy and Porosity.	↓	[113, 124]

**Table 3:** Process parameters.

Printing Parameter	Depiction	Description	Impact on dimensional accuracy identified in analysed studies from an increase (↑ top row) or decrease (↓ bottom row) in printing parameter value.	
Extruder temperature (ET)		The set temperature that melts the material so that it transitions from its solid state to a semi molten state for deposition. ET can also influence the defects listed: Circularity, Geometrical accuracy, Porosity, Surface Roughness, Stringing, and Warping.	↑	[28, 37, 67, 73, 77, 88, 105, 114, 118, 123, 125, 126, 127]
			↓	[34, 36, 38, 43, 54, 68, 83, 90, 92, 93, 95, 102, 108, 111, 112, 115, 116, 117, 128]
Nozzle diameter (ND)		The fixed diameter of the nozzle orifice, where the filament is extruded. This directly affects the road width. ND can also influence the defects listed: Geometrical accuracy and Surface Roughness.	**↑**	[6, 63]
			↓	[64, 112]
Bed temperature (BT)		The set temperature of the build envelope that enables adhesion between the material and substrate. BT can also influence the defects listed: Circularity, Geometrical accuracy, Shrinking, Surface Roughness, and Warping.	**↑**	[37, 114],
			↓	[54, 68]

**Table 4:** Design parameters.

Printing Parameter	Depiction	Description	Impact on dimensional accuracy identified in analysed studies from an increase (↑ top row) or decrease (↓ bottom row) in printing parameter value.	
Build orientation (BO)		The chosen orientation of a part on the build platform prior to printing. This affects an array of factors such as build time, mechanical properties, and support structures. BO can also influence the defects listed: Anisotropy, Circularity, Geometrical accuracy, Porosity, Surface Roughness, and Warping.	↑	[10, 33, 46, 71, 79, 81, 100, 129, 130]
			↓	[22, 32, 35, 44, 45, 60, 66, 69, 70, 72, 78, 85, 87, 96, 101, 110, 131, 132, 133, 134, 135]
Infill pattern (IP)		The internal architecture of the part, which affects mechanical properties, build time, printing cost, weight, and density. IP can also influence the defects listed: Geometrical accuracy and Surface roughness.		[33, 38, 39, 65, 77, 92, 95, 104, 122]
Infill density (ID)		The volume of material printed inside the part, which also affects mechanical properties, build time, printing cost, and weight. ID can also influence the defects listed: Circularity, Geometrical accuracy, Porosity, and Surface Roughness.	**↑**	[28, 36, 37, 54, 62, 65, 68, 69, 88, 97, 104, 108, 118, 124, 134]
			↓	[95, 96, 99, 109, 113, 128, 131, 133, 80, 93]
Build envelope position (BEP)		The location of the part on the build envelope.		[33, 107, 136]

**Table 5:** Environment and material choice.

Environment	Material	Printing Parameter	Impact on dimensional accuracy identified in analysed studies from an increase (↑ top row) or decrease (↓ bottom row) in printing parameter value	
With Enclosure	ABS	NGH	**↑**	
			↓	[12, 53, 54, 78, 93, 96, 103, 137]
	PLA	NGH	**↑**	[65, 67, 72, 75]
			↓	[89, 92, 98, 102, 108, 109]
Without Enclosure	ABS	NGH	**↑**	[10, 37, 76, 97]
			↓	[36, 87, 97, 104]
	PLA	NGH	**↑**	[32, 61, 97]
			↓	[31, 77, 82, 90, 91, 93, 94, 97, 100, 105, 107, 111, 112, 138]

### 4.1 Aspects of dimensional quality

The DQ of a component is an umbrella term which encompasses a range of specific measures, whereby an error in any one of these may be considered to represent a defect. Component defects are deviations of a produced part that haven't met the intrinsic requirements and/or nominal dimensions of the original virtual file. As previously stated, DA is one of the major aspects of DQ. DA refers to the difference between the measured part dimensions and the nominal model dimensions. By its very nature, this is one of the most significant defects and limitations of the ME AM process and an array of determinants can influence DA, primarily printing parameters. Due to the discontinuous nature of ME AM, defects such as DA are innate to the process and are recurrent [55]. Because of these inherent flaws, the ME AM process is somewhat impeded from producing ‘right first time’ end use components. The presence of these errors may potentially outweigh the benefits this technique possesses; however, efforts have been made in preventing and/or minimising these defects from transpiring [56].

It has been established from the systematic literature review, that printing parameters are known to significantly impact the DQ of printed components [12, 57, 58]. This will be discussed in greater detail in the subsequent sections. Modulation of any one of the ME AM machine's input variables will likely result in a change to the final part. For example, increasing the NGH, also known as layer height or layer thickness, can significantly affect DQ because of its layer by layer functionality intrinsic to the ME AM process [59]. This example demonstrates how important it is to consider the printing parameters and how they may play a significant role in reducing DQ error.

### 4.2 Printing parameter synthesis

The printing parameters can be classified into specific types. Turner and Gold have previously characterised the printing parameters into design, process, and toolpath parameters [23]. Table 2, Table 3, and Table 4 classify printing parameters, adapted from Turner and Gold's initial characterisation. Each table defines the printing parameter, the defects it can precipitate and the studies that have reported an improvement in part quality from an increase or decrease from the respective parameter value. If a study has determined that an increase in a printing parameter value has improved DA, said study will be in the top row with an upward arrow. Whereas, if a study has shown that a decrease in a printing parameter value has improved DA, it be in the bottom row with a downward arrow. For example, As NGH increases, studies including [10, 22, 25] etc. demonstrated an increase in DA. Conversely, studies [12, 26, 27] etc. demonstrated a decrease in DA.

Table 2, Table 3, and Table 4 demonstrate that for most printing parameters there was no clear consensus on whether a decrease or increase in parameter value led to an improvement in DA (and therefore in overall DQ). Thus, neither a positive nor negative correlation can be concluded for the majority of printing parameters. This suggests that part defects cannot be mitigated by the modulation of a single parameter alone and nor can a universal ‘one size fits all’ approach be used to improve part quality at present. Table 5, presents further analysis in how machine design, ABS and PLA coupled with NGH has an effect on DA. Similarly, no clear pattern was identified. NGH, PHV, RW, ET, BO, and ID have been thoroughly investigated as shown in Table 2, Table 3, and Table 4. Existing literature indicates that some of these parameters are more widely recognised to significantly influence defect types than others.

### 4.3 Dimensional accuracy

Of the 127 experimental studies reviewed in this study, 85 investigated DA. Across these studies, the relationship between all printing parameters listed in Table 2, Table 3, and Table 4 and DA have been explored. Despite this, no clear consensus has been demonstrated. However, a universal solution that considers material, machine, artefact design and printing parameters has yet to be proposed and in any case, would necessarily be very complex.

The first known published study (to the best of the authors knowledge) that experimentally investigated accuracies of an ME AM part was reported in 1995. This study compared the measured dimensions to the nominal values of a fabricated test artefact. The material used was P300 polymaide (Nylon), the NGH was 0.254 mm, which was the only parameter stated, and a coordinate measuring machine was used to characterise the test artefact. The results indicated that poor accuracy was realised, specifically for average X, Y, and Z distances of medium scale features. The authors speculated that this could have been a result of the course track used [139].

Subsequent to this publication, many studies have made efforts in addressing poor DA via parameter optimisation using a range of characterisation methods and statistical techniques. One of the most popular statistical methods employed to characterise the magnitude of response variables and realise the optimal parameter settings is the Taguchi method. Of the 127 papers reviewed in this study, 52 adopted this approach. Multiple studies [22, 28, 33] have demonstrated the use of this method. Of all the parameters investigated in Table 2, Table 3, and Table 4, five parameters have been shown to significantly influence DA, thus these parameters have been analysed in greater detail. Although printing parameters such as RA, CW, IP, and ID were considered in a few studies, there is limited evidence regarding their influence on DA and the evidence seems to indicate that they have little significance. For this reason, the remainder of our analysis will focus explicitly on the five most significant parameters.

### 4.4 Nozzle gap height

Erni et al., and Camposeco Negrete both employed the Taguchi method for determining the optimal parameters in reducing dimensional variation from the nominal values [33, 105]. It was concluded that a NGH of 0.1 mm and 0.3 mm respectively, improved dimensional accuracy. This suggests, that the smallest NGH does not consistently improve DA. Furthermore, both studies used alternative materials, machines, parameters, and test artefacts, which can all have an effect.

Typically, a reduced NGH is known for reducing the staircase effect as the height of each step is reduced, and the nominal geometry better followed in the build direction. However, it has not been demonstrated that this same relationship holds for DA improvement. Some studies have postulated that the test artefact global height must be an integer of the NGH to ensure minimal dimensional deviation in the Z axis [34, 95]. Moreover, when the NGH was 1⁄4 of the nozzle diameter, DA was improved; as opposed 1⁄2 the nozzle diameter, whereby gaps between layers were observed [91]. Ultimately, the NGH should not exceed 80% of the nozzle diameter [140]. Prior research has shown NGH's effect on DA further research is still required to optimise this parameter and its interaction effects.

### 4.5 Filament volumetric velocity

In addition to the Taguchi method, analysis of variance (ANOVA) coupled with signal to noise ratio (S/N Ratio) have been employed to determine the statistically significant parameters affecting the DA and measuring sensitivity of the quality investigated to enable respective parameter ranking [71]. Yadav et al., employed these methods, and thus identified that a print speed of 35 mm/s and a travel speed of 60 mm/s improved DA for a wax filament, whereas Abdul Haq et al., characterised that a print speed of 20 mm/s and a travel speed of 22 mm/s reduced dimensional error below 15% for a PCL/PLA composite [73]. This further demonstrates that neither a positive nor negative correlation between FVV and DA can be concluded.

One study investigated the flow tweak. A flow tweak (which changes material flow that induces percentage changes in strand width) when examined as an individual factor had an insignificant effect on shape fidelity when using the appropriate nozzle diameter for PLA. However, when presented as an interaction for all factor combinations using an artificial neural network, it was determined that FVV is significantly affected via NGH, flow tweak, PHV, and nozzle diameter, thus causing shape fidelity [100]. Therefore, importantly, these factors must be examined as a combination to produce reliable results.

It was also noted that higher filament volumetric velocities deposit excessive material at 90° corners at high print head velocities due to acceleration and deceleration as a result of motion changes [100]. Moreover, the extruder temperature has a significant effect on the material viscosity, causing over extrusion or under extrusion [114]. In order to optimise this parameter along with its effects, its interactions must be further investigated.

### 4.6 Print head velocity

In recent years, more advanced statistical techniques such as machine learning have been implemented to predict part quality characteristics. Sandhu et al., used a machine learning based prediction model to predict how PHV influences angular shrinkage of PLA parts [72]. The cross validation demonstrated that the predicted values were in good agreement with the experimental results, thus a PHV of 45 mm/s increased the DA. Shrinkage thus reduced as a result of increased PHV, as layout patterns are disturbed during slow deposition.

Durão et al., determined that a PHV of 30 mm/s reduced dimensional error with ABS. Furthermore, it was reported that PHV contributed 62.98% to model variance in the X and Y planes. Thus, this parameter should be kept at low values [36]. It is important to note, that in this study the interaction effects between PHV and the selected NGH were insignificant in the X and Y planes.

Rahman et al., employed Grey relational analysis coupled with the Taguchi method to perform a multi response analysis [54]. It was reported that a PHV of 55 mm/s improved dimensional accuracy of the ABS part while also considering the parameter interactions. An explanation as to why this value realised greater DA was not given.

One study performed manual measurements and determined the DA by subtracting the measured value from the nominal, without using statistical techniques. It was concluded that PHV did not have a significant effect on the DA using PLA. Furthermore, each parameter was characterised independently, thus ignoring their interactions. Additionally, the results presented that DA was constant for all PHVs except for a PHV of 90 mm/s. No clear trends could be established; however, it was postulated that this phenomenon might be a result of a heat transfer or a thermal gradient [92].

The studies reviewed present limited findings on the effect of PHV on DA and whether a high or low speed is recommended. However, it has been reported that a low speed can result in print deformation as the nozzle can physically interfere with the deposited material and a high speed can produce poor layer adhesion and insufficient cooling between layers [141]. Further work is required to fully understand how this parameter influences DA.

### 4.7 Build orientation

BO has been examined in many studies, as shown in Table 4. Although, this design parameter is known to have a significant effect on DA, this is largely dependent on the test artefact geometry, and the anisotropic effects as a result of the deposition process [66]. For example, Paul et al. [142], showed that build direction had a significant effect on the accuracy of a cylindrical test artefact. For more complex components with multiple features in a variety of directions, it may not be possible to align all features with their optimal build orientation. Regardless, a best practice guideline identified in prior studies, recommends that the DA is greatly improved if the test artefact is printed parallel to the X axis (0°) orthogonal to the Y axis (90°) or inversely [70, 135]. Alternatively, the part geometry will determine, which orientation it should be built in as the layer by layer deposition effects are inherent to this process.

### 4.8 Road width

The RW is a second order parameter and is a function of other primary printing parameters (such as ND, NGH, FVV, ET, and PHV), thus this has a significant influence on DA. An increase in RW generates a greater thermal gradient due to heat dissipation, which allows for shrinkage, thus enhancing DA [120]. One study reported that a lower RW can induce warping and deformation, reducing DA. Alternatively, if an increased RW is deposited, excess heat input is required, resulting in greater stress accumulation, and thus distorting the part. It was concluded that a low RW (0.4572 mm) was optimal for DA but was not a significant contributor [46]. However, Cruz Sanchez et al., reported that RW has major influence on DA and determined that a higher RW (0.71 mm) was optimal [61].

None of the above studies reported how the respective RWs were calculated, though it is most likely that this is conducted in the Slicer software. Knowledge of this calculation is fundamental in understanding the interaction effects between parameters. Moreover, importantly, this presents scope to investigate how each printing parameter affects the road width. One study proposed that the spatial fill can be calculated, whereby, “a strand cross sectional area has a width equal to the nozzle diameter and a height equal to the layer height”. The spatial fill can then be modulated via the FVV [143]. This spatial fill is effectively calculated in the Slicer Software.

### 4.9 Further analysis

The previous subsections clearly highlight that no clear consensus can be concluded in whether a printing parameter has a negative or positive relationship on DA. Table 5 analyses how the environment coupled with different materials and NGH can influence DA. Machine designs were distinguished between those with and those without enclosures. Statistically, more studies demonstrated that a decrease in NGH for both environments and materials did improve DA. However, this cannot be generalised, as some studies reported that an increase in NGH had improved DA. The findings from the studies that used an enclosure with ABS showed that a decrease in NGH explicitly improved DA. As ABS is highly sensitive to temperature changes, it is likely that the enclosure ensured temperature consistency and prevented heat loss from the build envelope [144, 145]. In contrast, the studies that did not use an enclosure with ABS showed that both an increase and decrease in NHG improved DA.

In summary, despite the large number of studies which have investigated parameter optimisation for the improvement of DA, no clear consensus exists on which are most important and what their respective values should be. This suggests that optimisation is a complex issue and is subject to the machine and material used and the artefact being produced. Therefore, there is the need for further research to improve DA.

## 5 Discussion

In this review, the authors undertook a holistic approach in reviewing ME AM experimental studies that have investigated the DQ of printed parts as a function of printing parameter modulation. The authors conducted a rigorous literature review, whereby 127 studies were critically analysed as shown in Appendix A, thus determining the relationships between printing parameters and DQ errors that have been demonstrated via experimental work. As a result of this analysis, no clear consensus has been identified for any of the most important printing parameters. Many studies came to contradictory conclusions for individual optimal parameters, even where artefact designs were similar. It is noted that despite the large body of work that has been reviewed, current machines and slicing software packages do not typically utilise their findings. Across the studies included in this review many machine, material, slicer, and artefact combinations have been used to determine the optimum set of printing parameters. The lack of agreement in optimum parameter values suggests that these combinations also have an influence and should be further investigated. Without this, the current literature forms only a partial solution to DA improvement. Due to the fact that the previous experimental studies can't be generalised, it is unknown how applicable this work is for future parameter optimisation studies. Adopting the recommendations from an individual existing study may negatively affect the results of future studies if the experimental set up is not precisely adhered to.

In this section, the authors first discuss the limitations of analysis techniques. Subsequently, the need for a standardised approach to better understand the influence of different machines, materials, and artefact geometries is examined. The benefits of understanding the morphology of the individual deposited strands are then proposed. Finally, the authors discuss the scope for future research.

### 5.1 Advanced techniques to characterise the printing parameter effects

There are almost an infinite number of machines, materials, artefact geometries, and printing parameter combinations that can be selected. As a result, each individual experiment, although rigorous on its own terms, only produces an answer which is accurate for the specific combination of parameters used. This significant complexity is not currently reflected in the parameter optimisation approaches in the literature. In order to best improve the DA of printed parts, an approach which captures this inherent complexity is required. Future studies would be greatly enhanced if they were to adopt more consistent approaches to experimental design in order for there to be improved generalisability.

Currently it is recognised that printing parameter selection is of significant importance to this process as noted by ISO ANSI (International Organization for Standardization American National Standards Institute) standards [13]. Various studies have investigated the effects of printing parameters albeit, each parameter was systematically isolated and subsequently examined [61, 92, 120]. Adopting this systematic approach as opposed to analysing the combination of printing parameters does not properly represent any interaction effects. ME AM requires each printing parameter to work in combination in order to print high quality parts. Thus, to ensure accurate results, the parameters must be analysed collectively.

A range of design of experiment techniques coupled with statistical optimisation methods have been used to derive the interaction effects of printing parameter. Mohamed et al., and Jaisingh Sheoran and Kumar both present thorough reviews of the experimental designs and optimisation techniques that have the ability to study the interaction effects between variables [146, 147]. However, both reviews proposed that better optimisation techniques need to be developed in order to address physical constraints within the process. In order to move towards a more rigorous representation of the array of independent variables possible within ME AM, new analysis techniques such as advanced mathematical modelling or machine learning technology is likely to be required.

### 5.2 Standardised approach of experimental methods to achieve generalisability

There is a lack of standardisation across the 127 studies analysed in this review. As discussed in section 3, studies were conducted on a wide range of machines, materials, and artefacts. This makes it challenging to compare studies directly and thus recommend a comprehensive set of parameters to optimise the DA of produced parts. The variety of optimal printing parameter values suggests that unique solutions exist for each combination of machine, material, and artefact. It is therefore important to better explore the effects of each of these on DA.

Currently, little is known about the specific effects of machine type on DA with very few studies using different machine types. Similarly, the effect of component geometry on the achievable DA has not been well covered in extant literature. However, it is more widely documented that different conventional thermoplastics require different printing parameters including that the printing parameters vary for different blends of the same thermoplastic. Polypropylene for example: the extrusion temperature can range from 165–250 degrees Celsius; NGH can vary between 0.1 mm to 0.35 mm, although this is also a function of artefact geometry [148].

The DA of the ME AM produced parts is highly dependent on the aforementioned variables. When characterising the DA of printed parts as a function of the less well explored machine, material, and artefact variables new studies which hold every other variable constant will prove beneficial and will ensure generalisability. For example, if one is investigating the effect of machine selection on DA: the material; artefact design; and printing parameters must remain unchanged on all the machines used in the experiment. Currently, the lack of standardisation across parameter optimisation studies makes such a comparison difficult. This targeted approach will provide greater insights into how poor DA is precipitated.

### 5.3 Implicit assumptions in slicing software

Significant efforts have been undertaken in investigating how the DA of produced parts could be improved via parameter optimisation experimentation. It can be determined that there is no clear consensus as to which combination of printing parameters can achieve this. As discussed, this is most likely a result of the inconsistent experimental methods that have been used between studies. One potential contributor to this lack of consensus is the implicit assumptions in the slicer software. Each Slicer has its own algorithm for generating the nozzle toolpath. As part of this, an effective strand width is assumed which can vary between slicers as well as depending on layer height and volumetric flow rate. If this is not controlled for, the use of different slicing software can therefore produce contradictory results and dimensional inaccuracies in artefacts. As a result, the findings from parameter optimisation studies cannot be generalised.

The relationships between slicer calculations and printing parameters must be characterised in order to account for the implicit assumptions in the software. Consequently, this could facilitate standardisation between future studies, with future experimentation being undertaken at a local strand level. Subsequent parameter optimisation studies without this generalisation are potentially unlikely to achieve impactful advancements. Thus, it is recommended that future efforts need to focus on understanding the process in greater detail, improving the underlying assumptions, and standardising the experimental methods initially at a local level.

### 5.4 Single strand morphologies

The studies analysed in this review have all examined the parameter effects and their interactions on the DA of a complete test artefact. By characterising the respective artefacts, the studies were able to report findings on DA. The ultimate dimensional quality of the external surface of a component is determined by the outermost boundary of each layer. Whilst all studies reviewed have considered ‘macro’ level errors, these originate from this ‘micro’ source. Thus, it is of paramount importance to experimentally investigate this underlying behaviour and variability by characterising strand morphologies. Understanding the micro level filament geometry will enable more sophisticated representations within the slicing software and reduce the need for extensive parameter optimisation.

Landmark studies, published by the Technical University of Denmark have made efforts in experimentally characterising single strand cross sections [149, 150, 151]. Initially, the authors proposed computational fluid dynamic simulations to investigate the morphology of strand cross sections. They identified that strand cross sections can significantly vary from being almost cylindrical to a flat cuboid depending on the NGH and PHV, thus influencing geometrical accuracy.

Subsequently, Serdeczny et al., validate their numerical model via experimental characterisation of single strand cross sectional measurements. As NGH is reduced, the cross sectional strand shape perpendicular to the print direction transitions from an oval shape to a cuboid with rounded corners. A NGH of 0.4 mm and PHV of 1.0 mm/s produced a rectangular strand at a width of 2.4 mm and height 0.4 mm. Whereas, a NGH of 1.2 mm and PHV of 1.0 mm/s produced a rectangular strand at a width of 1 mm and height 0.8 mm. As the PHV was reduced, a larger amount of filament was deposited for a set FVV [151]. These results are statistically significant, as they demonstrate the local dimensional variance that can be achieved via parameter modulation, thus affecting overall global part quality and geometry.

Additionally, a joint study between the Universities of Bradford and Nottingham, also measured the cross sectional perimeters of single strands [152]. The authors varied the FVV, PHV, and NGH, similar findings to the former studies were recorded. It is to be noted that all these studies performed measurements only in the plane orthogonal to the print direction, on specific printers, and only single strands were deposited. This analysis demonstrates that the underlying functions of this process are complex and require further investigation.

Another joint study, this time between the Universities of Nottingham and Loughborough present a volume conserving model, which simulates ME AM deposition [153]. This model provides a more accurate representation of material deposition as it allows for filament spreading and widening. The model results were well aligned with the experimental results. This study demonstrates how the filament behaviour and strand morphology is significantly influenced by crossover points. It is to be noted that the past three analysed studies have included numerical models in their works. These models, provide a useful insight in how the filament can behave during deposition, however, they are not without their limitations. For example, they are seemingly only accurate during steady state and not at the starts and ends of strands during the acceleration and deceleration phases respectively.

A recent study by Golab et al., investigated the morphology of single strands along the length of the strand and multiple stands [154]. The underlying machine variability of a Prusa i3 MK3 Multi Material desktop 3D printer was shown to be in the order of ±30 microns (strand height) and ±75 microns (strand width). As opposed to 500 microns (height) and 2600 microns (width) when varying printing parameters for single strands. Multiple strands exhibited similar dimensional variations; however, the morphological defects were far more noticeable when using extreme printing parameters. Furthermore, morphological variations were observed at the beginning and ends of single and multiple stands.

The research on single and multiple strands for improving the global part DA through printing parameter optimisation is still at its nascent stage. However, the efforts made provide a promising foundation for multiple input variable optimisation at the micro scale.

### 5.5 Scope for future research

ME AM was first developed in the 1980s and has since seen a plethora in new ME AM machines, materials, and capabilities. New machines and components have subsequently improved the process; however, the parts can still exhibit poor DA. As ME AM is a relatively new manufacturing technology, this recent influx is somewhat expected. The first known published study to the best of the authors knowledge, that experimentally investigated accuracies of an ME AM part was reported in 1995 [139]. The results indicated that poor DA was realised in all three axes of medium scale features. Subsequent studies now use more sophisticated experimental techniques and provide greater insights, but further research is still required to address the challenges this technology faces.

In this review it has been demonstrated that extensive research has been undertaken to explore how the printing parameters affect part quality and DA. However, most of the studies out of the 127 reviewed, didn't consider the generalisability of their experimental methods and reported the dimensional and geometrical accuracy of a complete artefact and not the cause of the underlying variability. As an artefact is built in a layer by layer succession, it is fundamental to understand the local variation that can occur. Therefore, future research in experimentally characterising deposited stands of filament is an exciting and important area to investigate.

Few studies have made efforts in characterising the morphology of single strands as a function of printing parameter modulation. Particularly, cross sections along the length of the strand. However, there are still many areas to be investigated. To date, very few studies have investigated the cross section interactions of multiple strands [154, 155]. Furthermore, there has been little discussion of machine, slicer, and material impacts on strand morphology. Additional research in this area to gain a greater understanding of micro level geometries will enable more accurate software to be developed, thus improving the DA of ME AM.

Exploring the influence of machine, slicer, material, and artefact design using standardised experimental approaches will provide more information on the sources on DA error. Additionally, this principal should be applied to single stands. For future investigation of these aspects particularly the slicing software's approximations, the authors recommend the adoption of greater standardisation of research methods such that studies may be compared with one another and will subsequently ensure improved generalisability. The authors support the conclusion of Mohamed et al., that in order to facilitate this standardisation, an experimental framework with clear rules and guidelines would be beneficial [146].

A novel mathematical analysis technique in quantifying the interaction effects, needs to be developed to overcome the physical constraints of ME AM. The technique needs to be easily understood; have multi objective optimisation; high accuracy; the model dynamics need to both be linear and non linear; and most importantly it must have the ability to find interactions between variables. Furthermore, it must adequately deal with a large number of input variables at multiple levels.

In ME AM, there are multiple sources of error including machine design (e.g., poor alignment of axes) time effects (e.g., warping, shrinkage etc). However, errors are significantly influenced by the printing parameters, particularly at a local level. Deviations can occur in individual strand morphologies (e.g., strand road width, strand height, start and ends of strands). The deviations in strand morphology appear to be significant and are an inherent characteristic of the process. It is possible to reduce some of the effects of the variations in individual strand morphologies through optimising printing parameters. However, to ensure more significant improvements and to better understand the interactions between the molten thermoplastic and the nozzle. There may be opportunities to further investigate this critical aspect of the ME AM process.

Although, new designs are still in their nascent stage, the integration of in situ monitoring and/or image characterisation via machine learning is also an exciting prospect to improve DA dynamically.

### 5.6 Limitations of this study

This review specifically examines the DA of parts produced from the ME AM process, and not alternative AM technologies. Furthermore, this review undertakes a holistic approach and does not investigate an explicit material, printer design, or printing parameter combination. Both ABS [156] and PP [148] have been reviewed previously. In this review, the authors excluded non experimental studies, therefore, studies that used numerical models and/or simulations to characterise the DA of printed parts were not included in the review table [149, 150]. The authors explicitly focused on the DA defect type; thus, the authors did not rigorously investigate alternative defects caused by printing parameter selection. These have already been thoroughly investigated [14, 146, 148].

## 6 Conclusion

This review provides a detailed overview of the past 25 years of experimental studies that have investigated the DA of produced parts in ME AM. The authors reviewed and critically analysed 127 studies to evaluate which machines, materials, sample sizes, artefact designs, and printing parameters that have been used thus far. Furthermore, the authors synthesised which printing parameters have been shown to affect DA and their optimal values. This review has shown a lack of alignment in suggested parameter values in part due to the lack of standardisation and generalisability between studies and that parameter optimisation is intrinsically related to specific experimental methods. This does, however, reflect the complex nature of the machine, material, and artefact designs used during the ME AM process. There is seemingly a major disconnect between the work done and its dissemination into practice that users can adopt as a result of the poor level of generalisability. However, this review also provides a resource for users of ME AM to identify specific optimisation studies that may be relevant to their respective machine, material, and artefact set up.

Although there is a large amount of experimental work which characterises the DA of produced parts, the techniques employed during these would benefit from further development. In particular, the printing parameter interaction effects are not always considered when proposing parameter optimisation solutions. Similarly, many of these studies are physically constrained in their experimental scope. Ultimately, parameter optimisation studies are of use when the input assumptions aren't met in reality and/or there are inherent problems with the process itself.

In summary, some printing parameters have been shown to have a significant effect on the DA of produced parts and others less so. Typically, a reduced NGH will reduce the staircase effect, therefore the nominal geometry is better adhered to. However, this relationship is not the same for DA improvement as it has been demonstrated that both an increase and decrease in NGH has improved DA. The effect of FVV on DA is inconclusive as this parameter cannot be analysed in isolation as its interactions with PHV are critical. However, it has been widely acknowledged that a high FVV with a low PHV can deposit excessive amounts of material per unit length and the opposite for a low FVV with a high PHV, thus the ratios need to be optimised in order to ensure DA improvement. Furthermore, a high PHV can result in poor adhesion between layers whereas a low PHV can lead to the nozzle physically interfering with deposited material. BO has been shown to have a considerable effect on DA, however, this is largely dependent on the test artefact geometry. It is recommended that if the artefact is oriented parallel to the X axis (0°) orthogonal to the Y axis (90°) or inversely, then DA is improved. The ET has a large effect on DA, but if one does not deviate from the recommended material temperature settings provided by the supplier, then theoretically the DA should not be affected. RW has a significant effect on DA. However, it is a function of ND, NGH, FVV, ET, and PHV, which is calculated in the Slicer software. Therefore, making it difficult to presently recommend what the optimal value should be as there are many variable interactions to consider. This however presents plenty of scope to investigate its effects. Overall, no general agreement on which are the most important printing parameters has been concluded and what their respective values should be. Optimisation is a complicated problem and is dependent on the machine and material used and the artefact being produced.

ME AM is an inexpensive alternative to many conventional manufacturing techniques and has the ability to realise complex geometries that would otherwise be impossible to fabricate via traditional means. Numerous research groups are making significant advancements in addressing poor DA. ME AM is a sequential layer by layer process whereby the morphology of a produced part is significantly influenced at a local level. Recent efforts have been made in characterising single deposited strands of thermoplastic by modulating the printing parameters. Developments in this area have demonstrated that local dimensional variance can occur, although further research is still required. It is recommended that a future crowed sourced experimental study should be undertaken, whereby each study participant prints single strands of filament using a Slicer's default printing parameters. All independent variables besides machine type should remain constant. The heights, widths, and areas of deposited strands should be reported and compared in order to determine the local DA that may occur. Additionally, In order to achieve real world impact, addressing the assumptions in the slicing software would enable greater standardisation and generalisation amongst users. Improving the DA of ME AM will facilitate increased adoption of this technology for industry, research, and wider society.

## Declarations

### Author contribution statement

All authors listed have significantly contributed to the development and the writing of this article.

### Funding statement

This work was supported by the UK Engineering and Physical Sciences Research Council (EPSRC) through the EPSRC Centre in Ultra Precision Engineering (EP/K503241/1).

### Data availability statement

No data was used for the research described in the article.

### Declaration of interest’s statement

The authors declare no conflict of interest.

### Additional information

No additional information is available for this paper.

## Figures and Tables

**Table:** 

Study	Machine	Slicer	Materials Used	Optimisation Method/Technique or Tools	Independent Variables	Characterisation Method	Sample Size	Test Artefact	Dependent Variables	Relationship
[139]	FDM Statasys	N/A	PLA	Error distribution	N/A	Coordinate measuring machine (CMM)	N/A	A square benchmark model (240 × 240 mm) with surface features	Dimensional accuracy	N/A
			P200 Resin							
[79]	N/A	QuickSlice	N/A	Fractional factorial experiment and analysis of variance (ANOVA)	Nozzle gap height Road width Air gap Build orientation Extruder temperature	Contact type surface measurement system	21 samples	Triangular prisms	Surface roughness (SR)	Nozzle gap height ↓ (SR ++) Build orientation 70° (SR ++)
[25]	N/A	N/A	N/A	Taguchi method, Analysis of Variance (ANOVA) and signal to noise ratio (SNR)	Nozzle gap height Road width Print head velocity	Surtronic surface roughness measurement tester	18 Samples	N/A	Surface roughness (SR)	Nozzle gap height↑ (SR ++) Road width ↓ (SR +) Print head velocity ↑ (SR +)
[136]	Stratasys FDM2000	N/A	ABS	Measurement deviations	Part size Build envelope position	Coordinate measuring machine (CMM)	12 samples repeated 3 times	Benchmark part with various small and large features	Dimensional accuracy (DA)	Part size (DA +) Position in the build envelope (DA ++) Bed temperature
					Bed temperature					
[157]	FDM Maxum RP	N/A	N/A	Direct measurement	Build angle	N/A	1 sample	Twisted truncheon	Surface roughness (SR)	Build angle 0/90/180 (SR+)
[21]	Maxum RP Machine	N/A	ABS	Taguchi method, Analysis of Variance (ANOVA) and signal to noise ratio (SNR)	Contour width Road width Raster angle Air gap	Mitutoyo BH303 coordinate measuring machine (CMM)	9 samples	A square benchmark model (50 × 50 mm) with surface features	Dimensional accuracy (DA)	Contour width ↑ (GF ++) Road width ↓ (DA ++) (GF ++) Raster angle ↑ (SR ++) Air gap ↑ (SR ++)
									Geometric forms (GF)	
									Surface roughness (SR)	
[26]	FDM Maxum	N/A	ABS	Mean surface roughness from 100 trial measurements	Nozzle gap height Surface angle	Surftest Formtracer Mitutoyo Corp., 1997	2 test parts	Rod with square planes axially rotated incrementally	Surface roughness (SR)	Nozzle gap height ↓ (SR ++) Surface angle at 90° (SR ++)
[27]	N/A	N/A	ABS	Full factorial experimental plan	Tip size Road width Nozzle gap height	Non-contact optimal system Optimet MiniConoscan 3000, that combines a non-contact, single-point measuring sensor (ConoProbe 1000)	8 samples repeated 3 times	Square base prisms 18 × 18 × 18 mm	Surface roughness (SR)	Road width ↓ (SR ++) Nozzle gap height ↓ (SR ++)
[22]	Vantage SE	N/A	ABS	Taguchi method, Analysis of Variance (ANOVA), signal to noise ratio, Gray Relational Analysis	Nozzle gap height Build orientation Raster angle Road width Air gap	Mitutoyo vernier calliper	27 samples	Cuboid 80 × 10 × 4 mm	Dimensional accuracy (DA)	Nozzle gap height ↑ (DA +) Build orientation ↓ (DA ++) Raster angle ↓ (DA +) Road width ↑ (DA +) Air gap ↑ (DA +)
[12]	Prodigy Plus	N/A	ABS	Taguchi method, Analysis of Variance (ANOVA), signal to noise ratio	Nozzle gap height Road width Raster angle Air gap	Micrometer	18 Samples	Cylinder ⌀12.7 × 25.4 mm	Dimensional accuracy (DA) Surface roughness (SR)	Nozzle gap height ↓ (DA ++) (SR ++) Road width ↑ (DA +) (SR +) Raster angle ↓ (DA +) (SR +) Air gap ↑ (DA +) (SR +)
[39]	Prodigy Plus	N/A	ABS	Full factorial experimental plan	Nozzle gap height Contour width Infill density	Touch-probe type coordinate measuring machine (CMM) (LH40, Wenzel, UK)	6 samples	Cuboid base with extruded features on the top and side surfaces	Dimensional accuracy (DA)	Contour width ↑ (DA ++) Infill density ↓ (DA ++)
[60]	Vantage SE machine	Insight FDM Software	ABS	Taguchi method, Analysis of Variance (ANOVA), signal to noise ratio, Gray Relational Analysis	Nozzle gap height Build orientation Raster angle Road width Air gap	Mitutoyo vernier calliper	27 samples repeated 3 times	Cuboid with two holes 80 × 10 mm	Dimensional accuracy (DA)	Nozzle gap height ↑ (DA ++) Build orientation ↓ (DA ++) Raster angle ↑ (DA +) Road width ↑ (DA +) Air gap ↓ (DA +)
[121]	Prodigy Plus	N/A	ABS	Design of experiments, analysis of variance (ANOVA), and signal to noise ratio	Contour width Contour depth Road width Raster angle	Coordinate measuring machine (CMM)	8 samples repeated 3 times	Spiral curve	Dimensional accuracy (DA)	Contour width ↑ (DA ++) Contour depth ↓ (DA ++) Road width ↑ (DA +) Raster angle (DA ++)
[158]	Prodigy FDM100	N/A	ABS	Experimental Plan–Full factorial, Analysis Techniques–ANOVA	Build position	Coordinate measuring machine (CMM)	8	Cyclinder 100 mm tall with conical core	Dimensional Accuracy (DA)	Vertical orientation best for DA.
					Nozzle gap height					
					Build orientation					
[80]	Dimension BST 768	CatalystEx	ABS	Design of experiments	Nozzle gap height Infill density Support density Stratification angle	Taylor Hobson Talypro- file Plus roughness profilometer	40 samples	Modified cuboid	Surface Roughness (SR)	Nozzle gap height ↓ (SR +) Infill density ↓ (SR +) Support density ↓ (SR +) Stratification angle 0° (SR ++)
[159]	MEM-300	N/A	ABS	Experimental Plan–Taguchi, Analysis Techniques–S/N ratio	Filament width	N/A	9 samples	60 mm × 20 mm × 9 mm	Dimensional Accuracy (DA)	Filament width ↓ (DA+), Filament volumetric velocity ↓ (DA+), Nozzle gap height ↓ (DA+)
					Filament volumetric velocity					
					Nozzle gap height					
[160]	FDM 1650	QuickSlice	N/A	Experimental Plan–Taguchi, Analysis Techniques–S/N ratio, ANOVA, F-Test	Nozzle gap height	Mitutoyo BH303 CMM (DA), Taylor Hobson Surtronic 3P (SR)	27	Component with hole, cylinder and various parallel surfaces	Dimensional Accuracy (DA), Surface Roughness (SR)	Nozzle gap height ↓ (SR+) Various relationships for DA depending on specific geometry
					Road width					
					Air gap					
					Print head velocity					
[131]	N/A	N/A	N/A	Measurements and calculated percentage difference	Build orientation Infill density	Coordinate measuring machine (CMM)	3 samples	Cube 40 × 40 × 40 mm, Cylinder ⌀30 × 50 mm, pyramid 35 × 35 × 35 mm	Dimensional accuracy (DA)	Build orientation ↓ (DA +) Infill density ↓ (DA +)
[161]	N/A	CatalystEx	ABS	Optical measurements	Build orientation	3D Optical Scanner	5 samples repeated 3 times	Dog bone 150 × 20 × 4 mm	Dimensional accuracy (DA)	Build orientation (DA ++)
[132]	N/A	N/A	ABS	Manual and optical measurements	Build orientation	Coordinate measuring machine (CMM) and SEM	16 samples	Cuboid with surface features 60 × 21.6 × 5 mm	Dimensional accuracy (DA)	Build orientation (DA ++)
[162]	N/A	N/A	ABS	Experimental Plan–Design of experiments	Extruder temperature	Calliper/Observation	8 samples	Component with variable 'bridging' sections, external dimension 14 × 18 mm	Unsupported features/layer adhesion/Quality (Q)	Extruder temperature ↓ (Q+) Filament volumetric velocity ↑ (Q+) Print head velocity ↑ (Q+)
					Filament volumetric velocity					
					Print head velocity					
[81]	Stratasys Dimension Bst 768	N/A	ABS	Manual Measurements	Build orientation Nozzle gap height	Micrometer	80 samples	Hexagonal shape geometry	Dimensional accuracy (DA)	Build orientation 90° (DA ++) Nozzle gap height ↓ (DA +)
[61]	FoldaRap, a derivative version of the RepRap	Slic3r	PLA	Taguchi method, Analysis of Variance (ANOVA)	Nozzle gap height Road width Print head velocity	Mitutoyo vernier calliper	9 samples repeated 2 times	A square benchmark model with surface features	Dimensional accuracy (DA)	Nozzle gap height ↑ (DA +) Road width ↑ (DA +) Print head velocity ↑ (DA ++)
[78]	Julia 3D printer	Insight FDM Software	ABS	Taguchi method, Analysis of Variance (ANOVA) and signal to noise ratio (SNR)	Nozzle gap height Build orientation Raster angle	Digital calliper	9 samples	Cube 10 × 10 × 10 mm	Dimensional accuracy (DA)	Nozzle gap height ↓ (DA ++) Build orientation ↓ (DA +) Raster angle ↓ (DA +)
[82]	CubeX printer	Netfabb Basic	PLA	Descriptive statistics	Nozzle gap height	Digital calliper	3 samples	Dry skull with plastic teeth	Dimensional accuracy (DA)	Nozzle gap height ↓ (DA ++)
[62]	Dimension Elite Stratasys	N/A	ABS	Dimensional and surface roughness measurements.	Nozzle gap height Infill density	Coordinate measuring machine (CMM) and Talysurf CLI 1000 3D profiler	4 samples	Cuboid 20 × 20 × 10 mm	Dimensional accuracy (DA) Surface roughness (SR)	Nozzle gap height ↑ (DA ++) ↓ (SR +) Infill density ↑ (DA +) (SR ++)
[83]	RapMan 3.0	Axon 2	HIPS	Observation	Extruder temperature Road width	N/A	27 samples	Cube 30 × 15 mm	Dimensional accuracy (DA)	Extruder temperature ↓ (DA ++) Road width ↑ (DA +) Nozzle gap height ↓ (DA +)
[63]	Stratasys FDM 3000	N/A	ABS	Design of experiments and analysis of variance (ANOVA)	Road width Nozzle gap height Nozzle diameter	Digital microscope	8 samples repeated 3 times	Cuboid 18 × 18 × 8 mm	Dimensional accuracy (DA)	Road width ↓ (DA +) Nozzle gap height ↑ (DA +) Nozzle diameter ↑ (DA ++)
[84]	LulzBot TAZ 4	N/A	ABS	Optical measurements	Nozzle gap height Filament volumetric velocity	Tool microscope	N/A	Cube	Surface roughness (SR)	Nozzle gap height ↓ (DA ++) Filament volumetric velocity ↓ (DA ++)
[163]	FDM-Dimension sst 1200es	CatalystEx	ABS PLUS	Direct measurement	Build angle	Surface scanner MahrSurf MFW250	1 (36)	Square-section twisted truncheon–36 cuboids of 10 × 30 × 30 mm	Surface roughness (SR)	Build angle 0/90deg best
[138]	RepRap Prusa-Mendel I2	Cura	PLA	Experimental Plan–Full Factorial DOE, Analysis Techniques–ANOVA, F-Test, p-values	Nozzle height	3Shape D700 Laser Scanner	81	Component with variety of square, cylinder, cone, dome, hole features	Dimensional Accuracy (DA)	Print head velocity ↓ (DA+) Nozzle gap eight ↓ (DA+) Mid level of filament volumetric velocity (105%) is best
					Print head velocity					
					Filament volumetric velocity					
[64]	N/A	N/A	ABS	Taguchi method, Analysis of Variance (ANOVA) and signal to noise ratio (SNR)	Road width Nozzle gap height Nozzle diameter	N/A	8 samples	Cuboid 18 × 18 × 8 mm	Dimensional accuracy (DA)	Road width ↓ (DA ++) Nozzle gap height ↑ (DA +) Nozzle diameter ↓ (DA +)
[85]	Pramaan mini	Cura	Nylon618	Taguchi method, Analysis of Variance (ANOVA) and signal to noise ratio (SNR)	Nozzle gap height Build orientation Shell thickness	Digital calliper	9 samples	Cube 10 × 10 × 10 mm	Dimensional accuracy (DA)	Nozzle gap height ↓ (DA ++) Build orientation ↓ (DA +) Shell thickness ↑ (DA +)
[44]	Fortus 400	N/A	PC-ABS blend	I-Optimality Design, Analysis of Variance (ANOVA)	Nozzle gap height Air gap Raster angle Build orientation Road width Number of shells	Micrometer	10 samples	Cuboid 35 × 12.5 × 3.5 mm	Dimensional accuracy (DA)	Nozzle gap height ↓ (DA ++) Air gap ↑ (DA ++) Raster angle 90° (DA +) Build orientation 90° (DA ++) Road width ↓ (DA ++) Number of shells ↓ (DA ++)
[45]	Titan	N/A	PC-ABS blend	Taguchi method, Analysis of Variance (ANOVA) and response surface methodology	Nozzle gap height Road width Air gap Build orientation Raster angle	Digital calliper	8 samples	Cuboid with rounded corners	Dimensional accuracy (DA)	Nozzle gap height ↓ (DA ++) Road width ↑ (DA ++) Air gap ↓ (DA ++) Build orientation 30° (DA +) Raster angle 0° (DA +)
[53]	Proto Center 999	Kisslicer Pro	ABS	Taguchi design method and Grey Relational Analysis	Nozzle gap height Road width Print head velocity Support material density	Digital calliper	9 samples	M20 bolt	Dimensional accuracy (DA)	Nozzle gap height ↓ (DA ++) Road width ↑ (DA +) Print head velocity ↑ (DA +) Support material density (DA +)
[129]	FDM Mojo	N/A	ABS	Surface roughness measurements	Build orientation	Surface roughness tester	7 samples	Modified cuboid with surface features 45 × 25 × 18.23 mm	Surface Roughness (SR)	Build orientation 90° (SR ++)
[164]	Ultimaker 2	N/A	N/A	Experimental Plan–Fractional design, Analysis Techniques–ANOVA, p-values, Black/white box modelling	Nozzle gap height	N/A	82	Component with hole, curved surface and various parallel surfaces	Surface Roughness (SR), Dimensional Accuracy (DA)	N/A
					Infill density					
					Print head velocity					
					Extruder temperature					
					Bed temperature					
					Bottom layer velocity					
					Infill speed					
[165]	RepRap 3D Printer	N/A	N/A	N/A	Print orientation, Support Geometry	Observation	4	Screw component, 30 mm diameter, 18 mm pitch	Roundness, visual defects	Roundness is improved in the vertical orientation.
[65]	Makerbot Replicator Z18	Slic3r	PLA	Fuzzy logic, Taguchi design method and signal to noise ratio	Nozzle gap height Print head velocity Infill pattern Infill density Number of shells	Mitutoyo vernier calliper	27 samples	Cuboid 35 × 12.5 × 3.5 mm	Dimensional accuracy (DA)	Nozzle gap height ↑ (DA ++) Print head velocity ↑ (DA +) Infill pattern ↑ (DA ++) Infill density ↑ (DA +) Number of shells ↓ (DA +)
[66]	FORTUS 400	N/A	ABS	Fuzzy logic, Taguchi design method, Analysis of Variance (ANOVA) and response surface methodology.	Nozzle gap height Build orientation Raster angle Road width Air gap	Mitutoyo vernier calliper	27 samples repeated 3 times	Cuboid 80 × 10 × 4 mm	Dimensional accuracy (DA)	Nozzle gap height ↑ (DA +) Build orientation 0° (DA ++) Raster angle 0° (DA +) Road width ↑ (DA +) Air gap ↑ (DA +)
[86]	Fab@home	N/A	poly-caprolactone (PCL) and Human Foreskin Fibroblasts (HFF) cells	Analysis of Variance (ANOVA) and imageJ	Air gap Nozzle gap height Build direction	Optical microscope	9 samples repeated 3 times	Square grids configurations with pores	Uniformity (U)	Air gap ↑ (U +) Nozzle gap height ↓ (U +) Build direction X axis (U +)
[87]	Pramaan mini	Cura	ABS	Taguchi method, Analysis of Variance (ANOVA) and signal to noise ratio (SNR)	Nozzle gap height Build orientation Shell thickness	Digital calliper	9 samples	Cube 10 × 10 × 10 mm	Dimensional accuracy (DA)	Nozzle gap height ↓ (DA ++) Build orientation 30° (DA +) Shell thickness ↓ (DA +)
[119]	Vantage SE	N/A	ABS	Response surface method, face centred central composite design, Analysis of Variance (ANOVA)	Raster angle Air gap Road width	Digital calliper	20 samples	N/A	Dimensional accuracy (DA)	Raster angle 30° (DA ++) Air gap ↑ (DA +) Road width ↓ (DA +)
[88]	Open Source printer	N/A	PLA	Taguchi method, Analysis of Variance (ANOVA) and signal to noise ratio (SNR)	Nozzle gap height Raster angle Infill density Extruder temperature Print head velocity	Perthometer S2 PGK surface analyser	27 samples	Rectangular cuboid 20 × 20 × 5 mm	Surface roughness (SR)	Nozzle gap height ↓ (SR ++) Raster angle 30° (SR ++) Infill density ↓ (SR +) Extruder temperature ↑ (SR ++) Print head velocity ↑ (SR +)
[89]	Da Vinci 1.0A	Slic3r	PLA	3D acquisition, point cloud, and NURBS	Nozzle gap height	Triangulation-based 3D scanner	6 samples	Cylinder 30 mm x ⌀20 mm	Dimensional accuracy (DA) Cylindricity (CY) Circularity (CI)	Nozzle gap height ↓ (DA ++) (CY +) (CI ​++)
[90]	Prusa i3	Cura	PLA	Manual Measurements	Extruder temperature Print head velocity Nozzle gap height	Digital calliper assumed	18 Samples	Right angle part with various features extruded and embossed on said part 40 × 16 × 3.5 mm	Dimensional accuracy (DA)	Extruder temperature ↓ (DA ++) Print head velocity (DA +) Nozzle gap height ↓ (DA ++)
[91]	Hybrid machine	Slic3r	PLA	Manual Measurements	Nozzle gap height	Scanning electron microscope and digital calliper assumed	3 samples	Cube 15 × 15 × 10 mm	Dimensional accuracy (DA)	Nozzle gap height ↓ (DA ++)
[128]	Makerbot Replicator	N/A	ABS	Point cloud data, statistical design of experiments and response surface regression analysis	Infill density. Extruder temperature	Laser scanning NextEngine HD laser scanner	34 samples repeated 2 times	Based on the NAS 979 standard	Geometrical dimensioning and tolerancing (GD&T)	Infill density ↓ (GD&T ++) Extruder temperature ↓ (GD&T +)
[113]	Prusa I3 Hephestos	Slic3r & Cura	PLA	Taguchi method and Analysis of Variance (ANOVA)	Nozzle gap height infill density First layer speed Infill print head velocity	Micrometer	8 samples repeated 3 times	Cube 15 × 15 × 15 mm	Dimensional accuracy (DA)	Nozzle gap height (DA +) infill density ↓ (DA ++) First layer speed (DA +) Infill print head velocity ↓ (DA ++) Perimeter velocity ↓ (DA ++) Slicer software Slic3r (DA ++) Extruder temperature (DA +) Extrusion multiplier ↑ (DA ++)
					Perimeter velocity Slicer software Extruder temperature Extrusion multiplier					
[6]	N/A	N/A	PC	Taguchi method, Analysis of Variance (ANOVA) and signal to noise ratio (SNR)	Nozzle diameter Support structure Build orientation	Coordinate measuring machine (CMM)	24 samples	rectangular parallelepiped 44.88 × 40 × 80 mm	Dimensional accuracy (DA)	Nozzle diameter ↑ (DA ++) Support structure (DA +) Build orientation Z (DA +)
[92]	Makerbot Replicator 2×	N/A	PLA	Manual Measurements	Build orientation Print head velocity Extruder temperature Nozzle gap height Infill density Infill pattern	Digital calliper & micrometre	18 Samples	Dog bone 115 × 19 × 3.5 mm	Dimensional accuracy (DA)	Build orientation (DA ++) Print head velocity ↓ (DA +) Extruder temperature ↓ (DA ++) Nozzle gap height ↓ (DA ++) Infill density (DA +) Infill pattern (DA +)
[46]	N/A	N/A	PC-ABS blend	IV-Optimal Response Surface Methodology (RSM), analysis of variance (ANOVA)	Nozzle gap height Air gap Raster angle Build orientation Road width Number of contours	Micrometer	60 samples	Cuboid 35 × 12.5 × 3.5 mm	Dimensional accuracy (DA)	Nozzle gap height ↑ (DA ++) Air gap ↓ (DA ++) Raster angle ↓ (DA +) Build orientation ↓ (DA +) Road width ↓ (DA +) Number of contours ↑ (DA +)
[166]	FDM-Dimension sst 1200es	CatalystEx	ABS PLUS	Direct measurement, ANN algorithm, trial and error, evolutionary algorithm	Build angle	Surface scanner MahrSurf MFW250	2	Square-section twisted truncheon–cuboids of 10 × 30 × 30 mm	Surface roughness	Build angle 0/90/180 (SR+)
					Nozzle gap height					
[167]	Ultimaker	Cura	N/A	Direct measurement	Infill geometry infill thickness	Coordinate measuring machine (CMM)	11	9 component with 20 mm diameter hole, 2 component with 3 different diameter holes 10–20 mm	Dimensional Accuracy (DA)	Sun rays' design (DA+) Number of lines ↑ (DA+) Thicker lines ↑ (DA+)
[168]	MakerBot Replicator Fifth Generation	Makerbot Print	-	Full factorial	Print head velocity	Veho VMS-001 portable USB digital microscope, ViTiny VT-300 portable USB digital microscope	9	Embossing plates with line elements -0.2 mm–4 mm circular elements 0.2 mm–4 mm and square elements 0.2 mm–4 mm	Dimensional Accuracy (DA)	Print head velocity ↑ (DA+)
[93]	Flashforge Finder Ultimaker 2 go XYZ da Vinci 2.0 Duo	N/A	PLA PLA ABS	Taguchi method, mean square error, and multi-objective error function	Extruder temperature Nozzle gap height Infill density	Coordinate measuring machine (CMM)	8 samples for each 3D printer	Rectangular cuboid 96.52 × 106.68 mm with various extruded and cut on the surface	Dimensional accuracy (DA)	Extruder temperature ↓ (DA ++) Nozzle gap height ↓ (DA +) Infill density ↓ (DA +)
[77]	3DP WORKBENCH	Simplify3d	PLA	Taguchi design method	Bed temperature Extruder temperature Nozzle gap height Number of shells Infill density Print head velocity	Coordinate measuring machine (CMM)	18 Samples	ASTM D5418-07 cuboid 35 mm length, width 12.5 and height 3.5	Dimensional accuracy (DA)	Extruder temperature ↑ (DA ++) Nozzle gap height ↓ (DA ++) Number of shells ↓ (DA +) Infill Pattern (DA ++) Number of solid layers ↓ (DA +)
					Infill pattern Number of solid layers					
[28]	Makerbot Replicator 2×	Makerbot Print	ABS	Taguchi design method and Signal-to-Noise-Ratio (SNR)	Chamber temperature	Coordinate measuring machine (CMM)	27 samples	A square benchmark component (80 × 80 mm) with 17 extruded and embossed features on the surface	Dimensional accuracy (DA)	Chamber temperature ↓ (DA ++) Extruder temperature ↑ (DA +) Number of shells ↑ (DA ++) infill shell spacing multiplier ↓ (DA ++) Inset distance multiplier ↓ (DA ++) Infill density ↑ (DA ++)
					Extruder temperature					
					Number of shells					
					Infill shell spacing multiplier					
					Inset distance multiplier					
					Infill density					
[54]	Proto Center 999	Kisslicer Pro	ABS	Taguchi design method and Grey Relational Analysis	Bed temperature Nozzle temperature Print head velocity Nozzle gap height Part infill number of loops	Coordinate measuring machine (CMM) & SURFTEST SJ 210	27 samples	Standard test bar with 6 stair step heights.	Dimensional accuracy (DA)	Bed temperature ↓ (DA/SR ++) Nozzle temperature ↓ (DA/SR +) Print head velocity ↑ (DA/SR +) Infill ↑ (DA/SR +) Nozzle gap height ↓ (DA/SR ++) Loops ↓ (DA/SR)
									Surface roughness (SR)	
[94]	Creality 3D CR-10	Simplify3d	PLA	Analysis of Variance (ANOVA) and imageJ	Nozzle gap height	Optical microscope	3 samples	M10 × 1.5 screws	Dimensional accuracy (DA)	Nozzle gap height ↓ (DA ++)
[95]	Commercial FDM 3D printer	N/A	PLA	Taguchi method, Analysis of Variance (ANOVA) and signal to noise ratio (SNR)	Extruder temperature Nozzle gap height Infill pattern Infill density	Digital calliper	9 samples repeated 3 times	Based on the ASTM D638-15 standard, 100 × 19 × 6 mm	Dimensional accuracy (DA)	Extruder temperature ↓ (DA ++) Nozzle gap height ↓ (DA ++) Infill pattern (DA) Infill density ↓ (DA ++)
[125]	Delta type spatial 3D printer	N/A	PLA	Cross section analysis	Extruder temperature	Optical microscope	2 samples	Vertical thin walls 40 × 1 × 20 mm	Porosity (P)	Extruder temperature ↑ (P ++)
[130]	Raise 3D N1 series 3D	N/A	PLA	Vision measurements	Build orientation	Rapid-I Vision Measuring System	5 samples	Rectangular block	Dimensional accuracy (DA)	Build orientation ↑ (DA ++)
[67]	Gimax3D S2	Slic3r	PLA	Taguchi design method	Extruder temperature Nozzle gap height Extruder multiplier Perimeter speed Small perimeter speed	Digital calliper	10 samples repeated 3 times	Cuboid 70 × 48 × 4 mm with geometric features extruded and cut on the surface	Dimensional accuracy (DA)	Extruder temperature ↑ (DA ++) Nozzle gap height ↑ (DA ++) Extruder multiplier ↑ (DA ++) Perimeter Speed ↓ (DA ++) Small perimeter speed ↓ (DA +)
[68]	ProtoCentre 999	Kisslicer Pro	ABS	Taguchi method, signal to noise ratio (SNR), and Grey Relational Analysis.	Bed temperature Extruder temperature Print head velocity Infill density Nozzle gap height Number of loops	Coordinate measuring machine (CMM) and Surftest SJ-210	27 samples	Grinder blade ⌀79 mm × 25 mm	Circularity (CI) Surface roughness (SR)	Bed temperature ↓ (CL ++) (SR ++) Extruder temperature ↓ (CL +) (SR +) Print head velocity ↓ (CL +) (SR +) Infill density ↑ (CL +) (SR +) Nozzle gap height ↑ (CL +) (SR +) Number of loops ↑ (CL ++) (SR ++)
[114]	N/A	Cura	Wax filament	Systematic optical measurements	Extruder temperature Print head velocity. Bed temperature Filament volumetric velocity	Optical microscope	24 samples	Wax cylinder pattern	Surface roughness (SR)	Extruder temperature ↑ (SR +) Print head velocity ↑ (SR +) Bed temperature ↑ (SR ++) Filament volumetric velocity ↑ (SR ++)
[69]	N/A	N/A	ABS	Taguchi design method and Analysis of Variance (ANOVA)	Nozzle gap height Infill density Build orientation	MITUTOYO SJ-301 surface roughness tester	9 samples	Cuboid 30 × 30 × 20 mm with a vertical hole	Surface roughness (SR)	Nozzle gap height ↑ (SR ++) Infill density ↑ (SR +) Build orientation YZ0˚ (SR ++)
[96]	Up-Mini	N/A	ABS	Taguchi design method and Analysis of Variance (ANOVA)	Nozzle gap height Infill density Inclination Build orientation	Digital calliper	18 Samples	Cuboid 15 × 15 × 10 mm	Dimensional accuracy (DA)	Nozzle gap height ↓ (DA ++) Infill density hollow (DA +) Inclination 0° (DA ++) Build orientation 0° (DA +)
[43]	Flashforge Finder	Cura	PLA/Wood	Optical measurements	Extruder temperature	Optical microscope	6 samples	Test tube 120 × 15 × 8 mm	Dimensional accuracy (DA)	Extruder temperature ↓ (DA ++)
[97]	Mendel Max Kossel Mini Pursa i3	Repetier	ABS PLA	Measurement deviations	Nozzle gap height Infill density	Profile Projector DS600 Handheld roughness tester TR200	16 samples	Rectangular cuboid consisting of hemispheres, cubes cylinders, and slots.	Dimensional accuracy (DA) Surface roughness (SR)	Nozzle gap height ↓ (DA ++) (SR ++) Infill density ↑ (DA ++) (SR ++)
[98]	Raise3D N2plus	N/A	PLA	Manual Measurements	Nozzle gap height	Digital calliper	10 samples	Cylinder 15 mm x ⌀10 mm	Dimensional accuracy (DA)	Nozzle gap height ↓ (DA ++)
[31]	Monoprice Maker Select V2	Cura	PLA	Taguchi design method and Analysis of Variance (ANOVA)	Nozzle gap height Print head velocity	Digital calliper	9 samples repeated 3 times	Dog bone 90 × 10 4 mm	Dimensional accuracy (DA)	Nozzle gap height ↓ (DA ++) Print head velocity ↑ (DA ++)
[122]	WITBOX printer	Cura	PLA	Taguchi design method and Analysis of Variance (ANOVA)	Infill Pattern Road width	Mitutoyo Surftest SJ-210 profilometer	15 samples	Cylinder 40 mm x ⌀30 mm	Surface roughness (SR)	Infill Pattern (SR) Road width ↓ (SR ++)
[99]	N/A	N/A	PLA	Taguchi design method and Analysis of Variance (ANOVA)	Nozzle gap height Print head velocity Infill density	VK-X150 Laser microscope	8 samples repeated 3 times	Cuboid	Surface roughness (SR)	Layer thickness ↓ (SR ++) Print head velocity ↑ (SR +) Infill density ↓ (SR ++)
[70]	N/A	N/A	ABS PLA	Taguchi design method, Analysis of Variance (ANOVA), signal to noise ratio (SNR), grey relational grade.	Nozzle gap height Raster angle Build orientation	Micrometre	8 samples per material	Cuboid 35 × 12.5 × 3.5 mm	Dimensional accuracy (DA)	Nozzle gap height ↑ (DA +) Raster angle ↓ (DA ++) Build orientation 0°, 90° (DA ++)
[133]	uPrint SE	N/A	ABS plus	Taguchi method, Analysis of Variance (ANOVA) and signal to noise ratio (SNR)	Infill density Horizontal build orientation Vertical build orientation	Dial gauge	9 samples	Tubular sample ⌀45 × 15 mm	Circularity (CI)	Infill density ↓ (Cl ++) Horizontal build orientation ↓ (Cl ++) Vertical build orientation ↓ (Cl +)
[10]	Pramaan mini	Cura	Taulmann Nylon	Taguchi method, Analysis of Variance (ANOVA) and signal to noise ratio (SNR)	Nozzle gap height Build orientation Shell thickness	Digital calliper	9 samples	Cube 10 × 10 × 10 mm	Dimensional accuracy (DA)	Nozzle gap height ↑ (DA ++) Build orientation 15°, 30° (DA +) Shell thickness ↓↑ (DA +)
			ABS							
[118]	Ultimaker 3	Cura	TPU	Manual Measurements	Infill density Print head velocity Extruder temperature	Digital calliper	16 samples	Rings	Dimensional accuracy (DA)	Infill density ↑ (DA +) Print head velocity ↓ (DA ++) Extruder temperature ↑ (DA ++)
[134]	uPrint-SE	N/A	ABS	Taguchi design method and Signal-to-Noise-Ratio (SNR)	Build orientation Infill density	Mitutoyo SJ-210 roughness tester	9 samples	Bluetooth headset cover	Surface roughness (SR)	Build orientation ↓ (SR +) (DA ++) Infill density ↑ (SR +) (DA +)
[169]	Dimension Elite Stratasys	N/A	ABSplus-P430/SR10-P400SR Support	Experimental Plan–Full factorial, Analysis Techniques–ANOVA, F-test, p-values	Length	3D Systems 3D scanner	66	20 mm × 20 mm × 1.5 mm–140 mm × 60 mm × 5.5 mm	Warpage/deflection (D)	Length↓ (D++), Height (Intermediate value worst), LH ↓ (D+)
					Width					
					Height					
					Nozzle gap height					
[29]	Julia 3D Printer	N/A	ABS	Pareto analysis	Print head velocity	Observation	100	1inch × 1inch × 1inch cube	Print failures/bed adhesion	-
					Initial layer height, print bed surface					
[170]	N/A	N/A	ABS	Experimental Plan–Full Factorial	Build Orientation	Contact surface (Taylor Hubson)	15	20 mm × 20 mm × 10 mm	Surface roughness (SR)	"it is proposed that Layer thickness L2 = 0.2540 mm with orientation number 4 (X = 0°, Y = 0° and Z = 45°) can be considered as the highest quality of the prototype with minimum cost"
					Nozzle gap height					
[171]	Picaso 250 Designer Pro, Designer X Pro	N/A	PLA, ABS, Nylon, Nylon-C, PETG, PP	N/A	Extrusion multiplier	Pressure testing, SEM	N/A	Cylinder, Cube, Pyramid, Sphere, Cone, Joined cylinder/cone	Porosity (P)	Extrusion multiplier ↑ (P+)
					Wall geometry					
					Wall structure					
					Extruder temperature					
[100]	3D Systems CubeX	Kisslicer Pro	PLA	Taguchi method, Analysis of Variance (ANOVA) and signal to noise ratio (SNR)	Nozzle gap height Flow tweak Print head velocity Build orientation	IMetric™ IScan M300 3D scanner	27 samples	Rectangular prism 20 × 28 × 38 mm	Dimensional accuracy (DA)	Nozzle gap height ↓ (DA ++) Flow tweak ↑ (DA +) Print head velocity ↑ (DA ++) Build orientation ↑ (DA +)
[101]	Dimension SST 1200es	CatalystEx	ABS plus	Percentage volumetric error	Nozzle gap height Build orientation	Digital calliper assumed	40 samples	Similar to ASTM D638 test artefacts	Dimensional accuracy (DA)	Nozzle gap height ↓ (DA ++) Build orientation ↓ (DA +)
[36]	3D Cloner ST	Slic3r	ABS	Taguchi design method and Analysis of Variance (ANOVA)	Road width Extruder temperature Nozzle gap height Print head velocity Infill density Number of contours	Digital calliper	47 samples	Cube 10 × 10 × 10 mm	Dimensional accuracy (DA)	Road width ↓ (DA +) Extruder temperature ↓ (DA +) Nozzle gap height ↓ (DA +) Print head velocity ↓ (DA ++) Infill density ↑ (DA +) Number of contours ↓ (DA ++)
[102]	Profi3DMaker	N/A	PLA	Taguchi design method and Analysis of Variance (ANOVA)	Nozzle gap height Extruder temperature	Optical measuring SmartScope MVP 200	2 different samples repeated 5 times	Trapezium and cylinder	Dimensional accuracy (DA)	Nozzle gap height ↓ (DA +) Extruder temperature ↓ (DA ++)
[135]	Stratasys Mojo	N/A	PLA	Manual Measurements and surface roughness tests	Build orientation	Digital calliper Mitutoyo Surftest SJ-301	5 samples repeated 3 times	Complex specimen	Dimensional accuracy (DA) Surface roughness (SR)	Build orientation X0° Y0, X90°, Y0° (DA ​++)
[71]	Flash Forge guider 2	Flash Print	Machinable wax filament	Taguchi method, Analysis of Variance (ANOVA) and signal to noise ratio (SNR)	Build orientation Nozzle gap height Filament volumetric velocity Print head velocity	Digital calliper	9 samples repeated 2 times	Cuboid 15 × 15 × 10 mm	Dimensional accuracy (DA)	Build orientation 90° (DA +) Nozzle gap height ↑ (DA ++) Filament volumetric velocity ↑ (DA +) Print head velocity ↓ (DA +)
[103]	Up! Mini	N/A	ABS	Data digitisation manipulation	Nozzle gap height Filament volumetric velocity Build orientation	3D structured light scanner	24 samples	Prisms and cylinders	Dimensional accuracy (DA)	Nozzle gap height ↓ (DA +) Filament volumetric velocity ↓ (DA +) Build orientation horizontal (DA ++)
[120]	FDM Vantage SE	N/A	ABS	Response surface methodology and analysis of Variance (ANOVA)	Raster angle Air gap Road width	Digital calliper	20 samples	Cuboid 30 × 10.5 × 10.5 mm	Dimensional accuracy (DA)	Raster angle 30° (DA ++) Air gap ↓ (DA +) Road width ↑ (DA +)
[72]	Flash Forge Guide 2	N/A	PLA	Machine learning based prediction	Build orientation Nozzle gap height Print head velocity	Coordinate measuring machine (CMM)	20 samples repeated 5 times	Trapezium 3 variations	Dimensional accuracy (DA)	Build orientation 20° (DA ++) Nozzle gap height ↑ (DA +) Print head velocity ↑ (DA +)
[104]	Zortax M200 3D	N/A	ABS	Taguchi design method, Analysis of Variance (ANOVA), and Analysis of Means (ANOM)	Raster angle Nozzle gap height Infill density Infill pattern	Digital calliper	9 samples	Cuboid with cylinder on the top surface 35 × 15 × 10 mm	Dimensional accuracy (DA)	Raster angle 0° (DA ++) Nozzle gap height ↓ (DA ++) Infill density ↑ (DA ++) Infill pattern Octagonal (DA ++)
[73]	FlashForge creator pro 2016	N/A	PCL/PLA	Taguchi design method	Extruder temperature Nozzle gap height Filament volumetric velocity Print head velocity	Coordinate measuring machine (CMM)	20 samples	Cuboid 20 × 20 × 10 mm	Dimensional accuracy (DA)	Extruder temperature ↑ (DA ++) Nozzle gap height ↑ (DA +) Filament volumetric velocity ↓ (DA ++) Print head velocity ↑ (DA ++)
[38]	Makerbot	Makerbot Print	N/A	Taguchi design method	Extruder temperature Filament volumetric velocity Fan power Number of shells Infill pattern	Digital calliper assumed	16 samples	Cuboid with various features on top and bottom surfaces	Dimensional accuracy (DA)	Extruder temperature ↓ (DA +) Filament volumetric velocity ↑ (DA +) Fan power ↓ (DA +) Number of shells ↓ (DA ++) Infill pattern diamond (DA +)
[37]	Odyssey Designex ×2	Cura	ABS	Taguchi design method and Analysis of Variance (ANOVA)	Nozzle gap height Extruder temperature Print head velocity Infill density Bed temperature	Mitutoyo Flexible Measuring Machine (FMM)	19 samples	Cuboid base with extruded features on the top surfaces	Dimensional accuracy (DA)	Nozzle gap height ↑ (DA +) Extruder temperature ↑ (DA +) Print head velocity ↑ (DA +) Infill density ↑ (DA +) Bed temperature ↑ (DA ++)
[34]	3D Volumic Stream 30 Pro	N/A	Glass reinforced polyamide (GRPA) Kevlar reinforced polyamide (KRPA)	Manual Measurements	Nozzle gap height Print head velocity Extruder temperature	Micrometre	7 samples for each material repeated 3 times	Dog bone 150 × 20 × 4 mm	Dimensional accuracy (DA)	Nozzle gap height ↓ (DA ++) Print head velocity ↓ (DA +) Extruder temperature ↓ (DA +)
[35]	Blockstec Blocks One	N/A	PLA	Taguchi design method, Analysis of Variance (ANOVA), and response surface methodology	Nozzle gap height Print head velocity. Build orientation	N/A	15 samples	Cube 10 × 10 × 10 mm	surface roughness (SR)	Nozzle gap height ↓ (DA +) Print head velocity Build orientation ↓ (DA +)
[105]	Wanhao Duplicator 5S Desktop	N/A	PLA	Taguchi method, Analysis of Variance (ANOVA), signal to noise ratio, Gray Relational Analysis	Nozzle gap height Extruder temperature Filament volumetric velocity Perimeter speed	Digital calliper	27 samples	3D cube 20 × 20 × 20 mm	Dimensional accuracy (DA)	Nozzle gap height ↓ (DA ++) (SR `++) Extruder temperature ↑ (DA +) (SR +) Filament volumetric velocity ↑ (DA ++) (SR +) Perimeter Speed ↑ (DA ++) (SR +)
									Surface roughness (SR)	
[172]	Pramaan Mini	Cura	Nylon 618	Experimental Plan–Taguchi L9, Analysis Techniques–Fuzzy-M-COPRAS	Shell thickness	Digital calliper	9	10 mm × 10 mm × 10 mm cube	Dimensional Accuracy (DA)	Multiple outcomes simultaneously optimised
					Nozzle gap height					
					Build orientation					
[173]	MakerBot Replicator 2×	Makerbot Print	Polycaprolactone (PCL)	Principal Component Analysis (PCA), Summed Standard Deviation (SSD), T-tests, p-values	Extruder temperature, Bed temperature	Linkam Imaging Station	12	Lattice structures	Dimensional Accuracy (DA)	Print head velocity ↑(DA+) Road width ↓(DA+)
					Print head velocity					
					Road width					
[174]	Tarantula Prusa i3	Cura	PLA	Central composite deign Response surface method, ANOVA, F-Test, p-values, PSO, SOS	Nozzle gap height	Micro Topper digital microscope, Mitutoyo SV-C4500 Formtracer	46	125 mm × 12.7 mm × 3.2 mm	Surface Roughness (SR)	Nozzle gap height (SR++) Print head velocity (SR++) Outer Shell speed (SR+) Extruder temperature (SR+)
					Print head velocity					
					Extruder temperature					
					Outer shell speed					
[175]	Folger Tech FDM	Repetier	ABS	Full factorial, ANOVA, F-Test, p-value, Response Surface Methodology (RSM)	Ambient temperature	Mitutoyo Surftest SJ-301 portable surface tester, Zeiss Contura G2 CMM	29	Pyramid components	Dimensional Accuracy (DA), Surface Roughness (SR)	Nozzle gap height ↓ (SR+) Ambient temperature ↓ (DA+)
					Nozzle gap height					
					Build orientation					
[176]	N/A	N/A	Polymaker Polysmooth (Polyvinyl Butyral)	Experimental Plan–Full factorial, Analysis Techniques–ANOVA, F-test	Nozzle gap height	N/A	48	Cuboid component	Dimensional Accuracy (DA)	Print head velocity ↑ (DA+) Nozzle gap height ↓ (DA+)
					Print head velocity					
					Perimeter shells					
[30]	Stratasys Dimension SST 768	N/A	PA, ABS	Direct measurement	Build orientation	Contact measurement SURFTEST SJ-210 (Mitutoyo), Optical measurement ATOS Core 135	3	Gear component, tip diameter 100.679 mm	Dimensional Accuracy (DA), Surface Roughness (SR)	Print orientation (SR+)
					Material					ABS Material (DA+)
[177]	"Low cost FDM machine"	Cura	ABS	Experimental Plan–Taguchi, Analysis Techniques–ANOVA, S/N Ratio	Extruder temperature	Mitutoyo Quick Vision 404, Microtech M835 Optical Microscope	9	60 mm × 30 mm × 10 mm cuboid	Warpage/deflection (D)	Bed temperature (D+)
					Bed temperature, Print head velocity					Chamber temperature (D+)
					Chamber temperature					
[178]	Raise3D N2 plus	N/A	PLA	Analysis Techniques–ANOVA, F-Test, p-values, RSM, nondominated sorting genetic algorithm II	Nozzle diameter	TR300 roughness meter	50	N/A	Surface Roughness (SR)	Nozzle diameter↓ (SR+) Nozzle gap height ↓ (SR+) Print head velocity ↓ (SR+) Filament volumetric velocity ↓ (SR+)
					Extruder temperature					
					Filament volumetric velocity print head velocity					
					Nozzle gap height					
[115]	Katana Akimbo FDM printer	N/A	ABS PLA	Analysis of Variance (ANOVA), linear regression model	Extruder temperature Filament Volumetric velocity	Calliper	30 samples	strips 0.4 × 0.4	Dimensional accuracy (DA)	Print temperature PLA ↓ (DA +) Print temperature ABS (DA +) Extrusion velocity PLA ↓ (DA +) Extrusion velocity ABS ↓ (DA +)
[33]	Stratasys F270	N/A	ASA	Taguchi method, Analysis of Variance (ANOVA) and signal to noise ratio (SNR)	Nozzle gap height Infill pattern Build orientation Printing plane Build envelope position	Micrometre	27 samples repeated 3 times	Dog bone ASTMD638-14	Dimensional accuracy (DA)	Nozzle gap height ↑ (DA +) Infill pattern ↑ (DA ++) Build orientation 90° (DA ++) Printing plane XY (DA ++) Build envelope position middle (DA +)
[179]	Ultimaker 2+	Cura	PLA	Taguchi design method	Print head velocity Cooling conditions Plate temperature	Micrometre	9 samples	Tubular sample ⌀20 × 25 mm	Dimensional accuracy (DA)	Print head velocity (DA ++) Cooling conditions 50% (DA +) Plate temperature (DA ++)
[124]	Commercial FDM printer	N/A	SiC/Al₂O₃ reinforced LDPE	Taguchi design method and Signal-to-Noise-Ratio (SNR)	Infill density Infill angle Infill print head velocity	Digital calliper	9 samples	Cuboid 35 × 10 × 10 mm	Dimensional accuracy (DA)	Infill density ↑ (DA +) Infill angle 75° (DA ++) Infill print head velocity ↓ (DA +)
[123]	Ultimaker Original	Cura	PLA	Taguchi design method, Analysis of Variance (ANOVA), Analysis of Means (ANOM), grey relational grade	Wall thickness Extruder temperature	Micrometer Mitutoyo Surftest SJ-210	9 samples	Cuboid with central recess 30 × 30 × 15 mm	Dimensional accuracy (DA) Surface roughness (SR)	Wall thickness ↑ (DA +) Extruder temperature ↑ (DA ++)
[116]	Custom delta FDM printer	N/A	ABS	Taguchi design method	Print head velocity Chamber temperature Extruder temperature	N/A	9 samples	Cuboid 100 × 100 × 5 mm	Warpage (W)	Print head velocity ↑ (DA ++) Chamber temperature ↑ (DA ++) Extruder temperature ↓ (DA +)
[32]	Mono-price Maker Select V2	N/A	PLA	Design of experiments, analysis of variance (ANOVA, and grey relational analysis	Nozzle gap height Print head velocity Build orientation	Digital calliper	30 samples	Dog bone 90 × 10 × 4 mm	Dimensional accuracy (DA)	Nozzle gap height ↑ (DA ++) Print head velocity ↓ (DA +) Build orientation 0° (DA +)
[106]	Makerbot Replicator 2×	Cura	ABS	Response surface method, and Analysis of Variance (ANOVA)	Nozzle gap height Angle of inclination Print head velocity	Mitutoyo SJ 201P standard surface roughness measuring device	20 samples	Trapezium 3 variations	Surface roughness (SR) Shrinkage (SA)	Nozzle gap height ↓ (SR +) (SA ++) Angle of inclination ↑ (SR +) (SA +) Print head velocity ↓ (SR ++) (SA +)
[56]	Fortus 250 mc	N/A	ABS	Taguchi method, Analysis of Variance (ANOVA) and signal to noise ratio (SNR)	Raster angle Road width Air gap	Taylor- Hobson, Sutronic 25 surface roughness measuring device	9 samples repeated 2 times	Modified cuboid with hole 50 × 20 × 15 mm	Surface roughness (SR)	Raster angle ↓ (SR ++) Road width ↓ (SR +) Air gap ↑ (SR +)
[137]	2040 Delta FDM machine	Cura	ABS	ANOVA, RSM Multi-repsonse optimization/desirability function	Nozzle gap height	Mitutoyo Surftest SJ-310 Surface roughness machine, Digital calliper	50	Pyramid/conical component	Dimensional Accuracy (DA)	Nozzle gap height↓ (SR+) Build orientation (SR+) Nozzle gap height (DA+) Print head velocity (DA+) Build orientation (DA+) Wall thickness↑ (DA+)
					Print head velocity					
					Build orientation				Surface Roughness (SR)	
					Wall Thickness					
					Extruder temperature					
[126]	Ultimaker 3	Cura	Technomelt PA 6910	N/A	Extruder Temperature	Observation	5 samples	Dog bone	Dimensional Accuracy (DA)	Extruder temperature ↑ (DA +)
[74]	Hyrel 3D Hydra 16A printer	N/A	Ink mixture	Design of experiments (DoE), 2 full factorial experimental series, ANOVA	Nozzle Gap Height Filament volumetric velocity	Optical microscope	16 samples x 3 times	Multiple Strands	Dimensional Accuracy (DA)	Nozzle Gap Height ↑ (DA ++) Filament volumetric velocity (DA +) Print head velocity (DA ++)
					Print head velocity					
[127]	3D+++ Xperia	N/A	Polymer composite	Direct measurement	Extruder Temperature	Calliper/CMM	Total 7 samples, each sample had a unique material composition	20 × 20 × 20 cubes	Dimensional Accuracy (DA)	Extruder temperature ↑ (DA +)
[117]	Ultimaker	Cura	ABS PLA CPE	Analysis of Variance (ANOVA) and linear regression model	Extruder temperature Print head velocity	Image Processing	30 samples	Strips	Dimensional Accuracy (DA)	ABS Extruder temperature ↓ (DA ++) ABS Print head velocity ↑ (DA +) PLA Extruder temperature (DA +) PLA Print head velocity ↑ (DA +) CPE Extruder temperature ↓ (DA +) CPE Print head velocity ↑ (DA +)
[107]	WAN- HAO Duplicator 6	Cura	PLA	N/A	Nozzle gap height Build orientation Raster angle	Digital calliper	27 samples	Dog bone	Dimensional Accuracy (DA)	Nozzle gap height ↓ (DA ++) Build orientation Flat ↑ (DA +) Raster angel (DA +)
[108]	MakerBot z18	Makerbot Print	PLA	Design of experiments and analysis of variance (ANOVA)	Nozzle gap height Infill density extruder temperature print head velocity	Digital calliper	32 samples and 32 samples	Pump connector and door hinge	Dimensional Accuracy (DA)	Nozzle gap height ↓ (DA ++) Infill density ↑ (DA +) extruder temperature ↓ (DA +) print head velocity ↓ (DA ++)
[109]	Ultimaker S5	Cura	PLA	Taguchi design method, Analysis of Variance (ANOVA), signal to noise ratio (SNR), grey relational grade.	Nozzle gap height Infill density infill pattern print head velocity	N/A	27 samples repeated 2 times	Dog bone	Dimensional Accuracy (DA)	Nozzle gap height ↓ (DA ++) Infill density ↓ (DA +) infill pattern print head velocity ↑ (DA +)
[75]	Zaxe Z1 +	xDesktop	PLA	N/A	Nozzle gap height	Digital calliper	9 samples	Square	Dimensional Accuracy (DA)	Nozzle gap height ↑ (DA ++)
[110]	Fortus 400	Insight FDM Software	PC-ABS blend	integrated second-order definitive screening design (DSD) and an artificial neural network (ANN) are	Nozzle gap height Air gap Raster angle Build orientation Road width Number of perimeters	Digital calliper	16 samples	Cylinder 40 mm × ⌀10 mm	Dimensional Accuracy (DA)	Nozzle gap height ↓ (DA ++) Air gap ↑ (DA +) Raster angle ↓ (DA +) Build orientation ↓ (DA ++) Road width ↓ (DA +) Number of perimeters ↑ (DA +)
[111]	Ultimaker 2+	Cura	PLA	N/A	Nozzle gap height Extruder temperature	Digital calliper	2	Benchmark part with various small and large features	Dimensional Accuracy (DA)	Nozzle gap height ↓ (DA + Extruder temperature ↓ (DA ++)
[76]	Creality Ender3	N/A	ABS	Design of experiments	Infill Density Extruder temperature Print head velocity Nozzle gap height	CMM	8 samples	Benchmark part with various small and large features	Dimensional Accuracy (DA) Surface Roughness (SR)	Infill Density (DA +) (SR +) Extruder temperature
										(DA +) (SR +) Print head velocity ↓ (DA ++)
										↓ (SR ++) Nozzle gap height ↑ (DA ++)
										↓ (SR ++)
[112]	Sigma R19 printer	N/A	PLA	Adaptive neuro-fuzzy inference systems (ANFIS) models	Nozzle diameter Extruder temperature Nozzle Gap Height Print head velocity Extrusion Multiplier	Vision measuring machine	19 samples	Hemispherical cups ID 32 mm, OD 50 mm	Dimensional Accuracy (DA)	Nozzle diameter
										↓ (DA ++) Extruder temperature
										↓ (DA +) Nozzle Gap Height↓ (DA ++) Print head velocity ↓ (DA +) Extrusion Multiplier
										↓ (DA +)

## References

[1] Gardan J. (2016). Additive manufacturing technologies: state of the art and trends. Int. J. Prod. Res..

[2] Sherman L.M. (2018). https://www.ptonline.com/blog/post/market-trends-in-polymer-additive-manufacturing.

[3] Bochmann L., Bayley C., Helu M., Transchel R., Wegener K., Dornfeld D. (2015). Understanding error generation in fused deposition modeling. Surf. Topogr. Metrol. Prop..

[4] Wong K.V., Hernandez A. (2012). A review of additive manufacturing. ISRN Mech. Eng..

[5] Fitzharris E.R., Watt I., Rosen D.W., Shofner M.L. (2018). Interlayer bonding improvement of material extrusion parts with polyphenylene sulfide using the Taguchi method. Addit. Manuf..

[6] Reyes-Rodríguez A., Dorado-Vicente R., Mayor-Vicario R. (2017). Dimensional and form errors of PC parts printed via Fused Deposition Modelling. Procedia Manuf..

[7] Bähr F., Westkämper E. (2018). Correlations between influencing parameters and quality properties of components produced by fused deposition modeling. Procedia CIRP.

[8] Grames E. (2020). https://all3dp.com/2/fused-deposition-modeling-fdm-3d-printing-simply-explained/.

[9] Kruth J.P. (1991). Material incress manufacturing by rapid prototyping techniques. CIRP Ann.

[10] Vishwas M., Basavaraj C.K., Vinyas M. (2018). Experimental investigation using taguchi method to optimize process parameters of fused deposition modeling for ABS and Nylon materials. Mater. Today Proc..

[11] Song R., Telenko C. (2019). Causes of desktop FDM fabrication failures in an open studio environment. Procedia CIRP.

[12] Nancharaiah T., Ranga Raju D., Ramachandra Raju V. (2010). An experimental investigation on surface quality and dimensional accuracy of FDM components. Int. J. Emerg. Technol..

[13] Equbal A., Equbal M.A., Sood A.K., Pranav R., Equbal M.I. (2018). A review and reflection on Part Quality improvement of fused deposition modelled parts. IOP Conf. Ser. Mater. Sci. Eng..

[14] Vyavahare S., Teraiya S., Panghal D., Kumar S. (2020). Fused deposition modelling: a review. Rapid Prototyp. J..

[15] Noriega A., Blanco D., Alvarez B.J., Garcia A. (2013). Dimensional accuracy improvement of FDM square cross-section parts using artificial neural networks and an optimization algorithm. Int. J. Adv. Manuf. Technol..

[16] Tong K., Lehtihet E.A., Joshi S. (2004). Software compensation of rapid prototyping machines. Precis. Eng..

[17] Pham M.T., Rajić A., Greig J.D., Sargeant J.M., Papadopoulos A., McEwen S.A. (2014). A scoping review of scoping reviews: advancing the approach and enhancing the consistency. Res. Synth. Methods.

[18] Gopalakrishnan S., Ganeshkumar P. (2013). Systematic reviews and meta-analysis: understanding the best evidence in primary healthcare. J. Fam. Med. Prim. Care.

[19] Moher D., Liberati A., Tetzlaff J., Altman D.G. (2009). Preferred reporting items for systematic reviews and meta-analyses: the PRISMA statement. PLoS Med..

[20] Cruz Sanchez F.A., Boudaoud H., Camargo M., Pearce J.M. (2020). Plastic recycling in additive manufacturing: a systematic literature review and opportunities for the circular economy. J. Clean. Prod..

[21] Kumar Y.R., Rao C.S.P., Reddy T.A.J. (2008). A robust process optimisation for fused deposition modelling. Int. J. Manuf. Technol. Manag..

[22] Sood A.K., Ohdar R.K., Mahapatra S.S. (2009). Improving dimensional accuracy of Fused Deposition Modelling processed part using grey Taguchi method. Mater. Des..

[23] Turner B.N., Gold S.A. (2015). A review of melt extrusion additive manufacturing processes: II. Materials, dimensional accuracy, and surface roughness. Rapid Prototyp. J..

[24] Singh S., Singh G., Prakash C., Ramakrishna S. (2020). Current status and future directions of fused filament fabrication. J. Manuf. Process..

[25] Anitha R., Arunachalam S., Radhakrishnan P. (2001). Critical parameters influencing the quality of prototypes in fused deposition modelling. J. Mater. Process. Technol..

[26] Ahn D., Kweon J.-H., Kwon S., Song J., Lee S. (2009). Representation of surface roughness in fused deposition modeling. J. Mater. Process. Technol..

[27] Galantucci L.M., Lavecchia F., Percoco G. (2009). Experimental study aiming to enhance the surface finish of fused deposition modeled parts. CIRP Ann.

[28] Mahmood S., Qureshi A.J., Talamona D. (2018). Taguchi based process optimization for dimension and tolerance control for fused deposition modelling. Addit. Manuf..

[29] Devicharan R., Garg R. (2019). 3D Print. Addit. Manuf. Technol..

[30] Mašović R., Jagarčec V., Miler D., Domitran Z., Bojčetić N., Žeželj D. (2019).

[31] Velineni A., Günay E.E., Park K., Okudan Kremer G.E., Schnieders T.M., Stone R.T. (2018). An investigation on selected factors that cause variability in additive manufacturing. IISE Annu. Conf. Expo.

[32] Günay E.E., Velineni A., Park K., Okudan Kremer G.E. (2020). An investigation on process capability analysis for fused filament fabrication. Int. J. Precis. Eng. Manuf..

[33] Camposeco-Negrete C. (2020). Optimization of FDM parameters for improving part quality, productivity and sustainability of the process using Taguchi methodology and desirability approach. Prog. Addit. Manuf..

[34] Ouballouch A., El Alaiji R., Ettaqi S., Bouayad A., Sallaou M., Lasri L. (2019). Evaluation of dimensional accuracy and mechanical behavior of 3D printed reinforced polyamide parts. Procedia Struct. Integr..

[35] Castelão A., Soares B.A.R., Machado C.M., Leite M., Mourão A.J.M. (2019). Design for AM: contributions from surface finish, part geometry and part positioning. Procedia CIRP.

[36] Durão L.F.C.S., Barkoczy R., Zancul E., Lee Ho L., Bonnard R. (2019). Optimizing additive manufacturing parameters for the fused deposition modeling technology using a design of experiments. Prog. Addit. Manuf..

[37] Marwah O.M.F., Yahaya N.F., Darsani A., Mohamad E.J., Haq R.H.A., Johar M.A., Othman M.H. (2019). Investigation for shrinkage deformation in the desktop 3D printer process by using DOE approach of the ABS materials. J. Phys. Conf. Ser..

[38] Herath H.M.D.B., Thalagala S., Gamage P. (2019). Enhancing the dimensional accuracy of components fabricated using rapid prototyping technique by optimizing machine parameters of a 3D printer. 2019 IEEE Int. Conf. Ind. Eng. Eng. Manag..

[39] Bakar N.S.A., Alkahari M.R., Boejang H. (2010). Analysis on fused deposition modelling performance. J. Zhejiang Univ. A..

[40] Moylan J.S.S., Cooke A., Jurrens K., Donme M.A. (2012). Proposal for a standardized test artifact for additive, solid free. Fabr. Symp..

[41] de Macedo R.Q., Ferreira R.T.L., Gleadall A., Ashcroft I., Volco X. (2021). Numerical simulation of material distribution and voids in extrusion additive manufacturing. Addit. Manuf..

[42] Dey A., Hoffman D., Yodo N. (2020). Optimizing multiple process parameters in fused deposition modeling with particle swarm optimization. Int. J. Interact. Des. Manuf..

[43] Cekic A., Begic-Hajdarevic D., Muhamedagic K., Guzanovic N. (2018). Ann. DAAAM Proc. Int. DAAAM Symp..

[44] Mohamed O.A., Masood S.H., Bhowmik J.L. (2016). Optimization of fused deposition modeling process parameters for dimensional accuracy using I-optimality criterion. Measurement.

[45] Devika D., Gupta N.S. (2016). Modeling the process parameters of RP-FDM using ANOVA and response surface methodology for PC-ABS materials. 2016 Int. Conf. Electr. Electron. Optim. Tech., IEEE.

[46] Mohamed O.A., Masood S.H., Bhowmik J.L. (2017). Experimental investigation for dynamic stiffness and dimensional accuracy of FDM manufactured part using IV-Optimal response surface design. Rapid Prototyp. J..

[47] Das A., Gilmer E.L., Biria S., Bortner M.J. (2021). Importance of polymer rheology on material extrusion additive manufacturing: correlating process physics to print properties. ACS Appl. Polym. Mater..

[48] Sanchez L.C., Beatrice C.A.G., Lotti C., Marini J., Bettini S.H.P., Costa L.C. (2019). Rheological approach for an additive manufacturing printer based on material extrusion. Int. J. Adv. Manuf. Technol..

[49] Coogan T.J., Kazmer D.O. (2019). In-line rheological monitoring of fused deposition modeling. J. Rheol..

[50] Soares J.B., Finamor J., Silva F.P., Roldo L., Cândido L.H. (2018). Analysis of the influence of polylactic acid (PLA) colour on FDM 3D printing temperature and part finishing. Rapid Prototyp. J..

[51] Baumann F., Bugdayci H., Grunert J., Keller F., Roller D. (2016). Influence of slicing tools on quality of 3D printed parts. Comput. Aided. Des. Appl..

[52] Šljivic M., Pavlovic A., Kraišnik M., Ilić J. (2019). Comparing the accuracy of 3D slicer software in printed enduse parts. IOP Conf. Ser. Mater. Sci. Eng..

[53] Anusree T.G., Anjan R.N., Sivadasan M., John T.D. (2016). Process parameter optimization of fused deposition modeling for helical surfaces using grey relational analysis. Mater. Sci. Forum.

[54] Rahman H., John T.D., Sivadasan M., Singh N.K. (2018). Investigation on the scale factor applicable to ABS based FDM additive manufacturing. Mater. Today Proc..

[55] Batista M., Valerga A.P., Salguero J., Fernandez-Vidal S.R., Girot F. (2019). Addit. Subtractive Manuf., De Gruyter.

[56] Khan M.S., Mishra S.B. (2020). Minimizing surface roughness of ABS-FDM build parts: an experimental approach. Mater. Today Proc..

[57] Sun Q., Rizvi G.M., Bellehumeur C.T., Gu P. (2008). Effect of processing conditions on the bonding quality of FDM polymer filaments. Rapid Prototyp. J..

[58] Malekipour E., Attoye S., El-Mounayri H. (2018). Investigation of layer based thermal behavior in fused deposition modeling process by infrared thermography. Procedia Manuf..

[59] Pandey P.M., Venkata Reddy N., Dhande S.G. (2003). Improvement of surface finish by staircase machining in fused deposition modeling. J. Mater. Process. Technol..

[60] Sood A.K., Ohdar R.K., Mahapatra S.S. (2010). Parametric appraisal of fused deposition modelling process using the grey Taguchi method. Proc. Inst. Mech. Eng. Part B J. Eng. Manuf..

[61] Cruz Sanchez F.A., Boudaoud H., Muller L., Camargo M. (2014). Towards a standard experimental protocol for open source additive manufacturing. Virtual Phys. Prototyp..

[62] Nuñez P.J., Rivas A., García-Plaza E., Beamud E., Sanz-Lobera A. (2015). Dimensional and surface texture characterization in fused deposition modelling (FDM) with ABS plus. Procedia Eng..

[63] Galantucci L.M., Bodi I., Kacani J., Lavecchia F. (2015). Analysis of dimensional performance for a 3D open-source printer based on fused deposition modeling technique. Procedia CIRP.

[64] Dixit N.K., Srivastava R., Narain R. (2016). Comparison of two different rapid prototyping system based on dimensional performance using grey relational grade method. Procedia Technol.

[65] Huu Nghi Huynh, Anh Tuan Nguyen, Ngoc Luan Ha, Thi Thu Ha Thai (2017). 2017 Int. Conf. Syst. Sci. Eng., IEEE.

[66] Padhi S.K., Sahu R.K., Mahapatra S.S., Das H.C., Sood A.K., Patro B., Mondal A.K. (2017). Optimization of fused deposition modeling process parameters using a fuzzy inference system coupled with Taguchi philosophy. Adv. Manuf..

[67] Buonamici F., Carfagni M., Furferi R., Governi L., Saccardi M., Volpe Y. (2018). Optimizing fabrication outcome in low-cost FDM machines. Part 2–tests. Manuf. Technol..

[68] Sajan N., John T.D., Sivadasan M., Singh N.K. (2018). An investigation on circularity error of components processed on Fused Deposition Modeling (FDM), Mater. Today Proc.

[69] Chaudhari M., Jogi B.F., Pawade R.S. (2018). Comparative study of Part Characteristics built using additive manufacturing (FDM). Procedia Manuf..

[70] Hyndhavi D., Babu G.R., Murthy S.B. (2018). Investigation of dimensional accuracy and material performance in fused deposition modeling. Mater. Today Proc..

[71] Yadav A.C., Navin Kumar N., Raja K., Naiju C.D. (2019).

[72] Sandhu K., Singh S., Prakash C. (2019). Analysis of angular shrinkage of fused filament fabricated poly-lactic-acid prints and its relationship with other process parameters. IOP Conf. Ser. Mater. Sci. Eng..

[73] Abdul Haq R.H., Faizan Marwah O.M., Abdol Rahman M.N., Haw H.F., Abdullah H., Ahmad S. (2019). 3D Printer parameters analysis for PCL/PLA filament wire using Design of Experiment (DOE). IOP Conf. Ser. Mater. Sci. Eng..

[74] Jang S., Boddorff A., Jang D.J., Lloyd J., Wagner K., Thadhani N., Brettmann B. (2021). Effect of material extrusion process parameters on filament geometry and inter-filament voids in as-fabricated high solids loaded polymer composites. Addit. Manuf..

[75] Taşdemir V. (2021). Investigation of dimensional integrity and surface quality of different thin-walled geometric parts produced via fused deposition modeling 3D printing. J. Mater. Eng. Perform..

[76] Spindola-Filho J., Piratelli-Filho A., Arencibia R. (2020). CAD’20, CAD Solutions LLC.

[77] Hamza I., Abdellah E.G., Mohamed O. (2018). Experimental optimization of fused deposition modeling process parameters: a Taguchi process approach for dimension and tolerance control. Proc. Int. Conf. Ind. Eng. Oper. Manag. 2018.

[78] Nidagundi V.B., Keshavamurthy R., Prakash C.P.S. (2015). Studies on parametric optimization for fused deposition modelling process. Mater. Today Proc..

[79] Vasudevarao B., Natarajan D.P., Henderson M. (2000). Sensitivity of rp surface finish to process parameter variation, solid free. Fabr. Proc..

[80] Boschetto A., Giordano V., Veniali F. (2012). Modelling micro geometrical profiles in fused deposition process. Int. J. Adv. Manuf. Technol..

[81] Boschetto A., Bottini L. (2014). Accuracy prediction in fused deposition modeling. Int. J. Adv. Manuf. Technol..

[82] Petropolis C., Kozan D., Sigurdson L. (2015). Accuracy of medical models made by consumer-grade fused deposition modelling printers. Plast. Surg..

[83] Kaveh M., Badrossamay M., Foroozmehr E., Hemasian Etefagh A. (2015). Optimization of the printing parameters affecting dimensional accuracy and internal cavity for HIPS material used in fused deposition modeling processes. J. Mater. Process. Technol..

[84] Jin Y., Li H., He Y., Fu J. (2015). Quantitative analysis of surface profile in fused deposition modelling. Addit. Manuf..

[85] Basavaraj C.K., Vishwas M. (2016). Studies on effect of fused deposition modelling process parameters on ultimate tensile strength and dimensional accuracy of Nylon. IOP Conf. Ser. Mater. Sci. Eng..

[86] Ceretti E., Ginestra P., Neto P.I., Fiorentino A., Da Silva J.V.L. (2017). Multi-layered scaffolds production via fused deposition modeling (FDM) using an open source 3D printer: process parameters optimization for dimensional accuracy and design reproducibility. Procedia CIRP.

[87] Vishwas M., Basavaraj C.K. (2017). Studies on optimizing process parameters of fused deposition modelling technology for ABS. Mater. Today Proc..

[88] Sukindar N.A., Ariffin M.K.A.M., Baharudin B.T.H.T., Jaafar C.N.A., Ismail M.I.S. (2017). Optimization of the parameters for surface quality of the open-source 3D printing. J. Mech. Eng. SI.

[89] Ingrassia T., Nigrelli V., Ricotta V., Tartamella C. (2017). Lect. Notes Mech. Eng..

[90] Polak R., Sedlacek F., Raz K. (2017). Ann. DAAAM Proc. Int. DAAAM Symp..

[91] Tomal A.N.M.A., Saleh T., Khan M.R. (2017). Improvement of dimensional accuracy of 3-D printed parts using an additive/subtractive based hybrid prototyping approach. IOP Conf. Ser. Mater. Sci. Eng..

[92] Alafaghani A., Qattawi A., Alrawi B., Guzman A. (2017). Experimental optimization of fused deposition modelling processing parameters: a design-for-manufacturing approach. Procedia Manuf..

[93] Lyu J., Manoochehri S. (2018). Modeling machine motion and process parameter errors for improving dimensional accuracy of fused deposition modeling machines. J. Manuf. Sci. Eng..

[94] Tronvoll S.A., Elverum C.W., Welo T. (2018). Dimensional accuracy of threads manufactured by fused deposition modeling. Procedia Manuf..

[95] Alafaghani A., Qattawi A. (2018). Investigating the effect of fused deposition modeling processing parameters using Taguchi design of experiment method. J. Manuf. Process..

[96] Haghighi A., Li L. (2018). Study of the relationship between dimensional performance and manufacturing cost in fused deposition modeling. Rapid Prototyp. J..

[97] Ramli F.R., Faudzie M.S.M., Nazan M.A., Alkahari M.R., Sudin M.N., Mat S., Khalil S.N. (2018). Dimensional accuracy and surface roughness of part features manufactured by open source 3D printer. ARPN J. Eng. Appl. Sci..

[98] Wu J. (2018). Study on optimization of 3D printing parameters. IOP Conf. Ser. Mater. Sci. Eng..

[99] Peng T., Yan F. (2018). Dual-objective analysis for desktop FDM printers: energy consumption and surface roughness. Procedia CIRP.

[100] Papazetis G., Vosniakos G.-C. (2019). Mapping of deposition-stable and defect-free additive manufacturing via material extrusion from minimal experiments. Int. J. Adv. Manuf. Technol..

[101] Dambatta Y.S., Sarhan A.A.D., Maher I., Hourmand M. (2019). Volumetric shrinkage prediction in fused deposition modelling process–ANFIS modelling approach. Int. J. Mater. Prod. Technol..

[102] Beniak J., Križan P., Šooš Ľ., Matúš M. (2019). Research on shape and dimensional accuracy of FDM produced parts. IOP Conf. Ser. Mater. Sci. Eng..

[103] Mora S.M., Gil J.C., Camacho López A.M. (2019). Influence of manufacturing parameters in the dimensional characteristics of ABS parts obtained by FDM using reverse engineering techniques. Procedia Manuf..

[104] Tsiolikas A., Mikrou T., Vakouftsi F., Aslani K.E., Kechagias J. (2019). Robust design application for optimizing ABS fused filament fabrication process: a case study. IOP Conf. Ser. Mater. Sci. Eng..

[105] Erni D., Wiesmann D., Spühler M., Hunziker S., Moreno E., Oswald B., Fröhlich J., Hafner C. (2019). Application of taguchi method in the optimization of 3D-printer process parameters for dimensional accuracy and surface roughness. J. Manuf. Technol. Res..

[106] Eswaran P., Subramaniyan M., Appusamy A., Annakalyani N.S., Pusapati S.R. (2020). Mater. Today Proc..

[107] Hanon M.M., Zsidai L., Ma Q. (2021). Accuracy investigation of 3D printed PLA with various process parameters and different colors. Mater. Today Proc..

[108] Galetto M., Verna E., Genta G. (2021). Effect of process parameters on parts quality and process efficiency of fused deposition modeling. Comput. Ind. Eng..

[109] Enemuoh E.U., Duginski S., Feyen C., Menta V.G. (2021). Effect of process parameters on energy consumption, physical, and mechanical properties of fused deposition modeling. Polymers.

[110] Mohamed O.A., Masood S.H., Bhowmik J.L. (2021). Modeling, analysis, and optimization of dimensional accuracy of FDM-fabricated parts using definitive screening design and deep learning feedforward artificial neural network. Adv. Manuf..

[111] Syrlybayev D., Perveen A., Talamona D. (2021). Fused deposition modelling: effect of extrusion temperature on the accuracy of print. Mater. Today Proc..

[112] Luis-Pérez C.J., Buj-Corral I., Sánchez-Casas X. (2021). Modeling of the influence of input AM parameters on dimensional error and form errors in PLA parts printed with FFF technology. Polymers.

[113] Santana L., Lino Alves J., da Costa Sabino Netto A. (2017). A study of parametric calibration for low cost 3D printing: seeking improvement in dimensional quality. Mater. Des..

[114] Kuo C.-C., You Z.Y. (2018). A cost-effective approach for rapid fabricating cooling channels with smooth surface. Int. J. Adv. Manuf. Technol..

[115] Akbaş O.E., Hıra O., Hervan S.Z., Samankan S., Altınkaynak A. (2019). Dimensional accuracy of FDM-printed polymer parts. Rapid Prototyp. J..

[116] Zhou Y., Lu H., Wang G., Wang J., Li W. (2020). Voxelization modelling based finite element simulation and process parameter optimization for Fused Filament Fabrication. Mater. Des..

[117] Hıra O., Yücedağ S., Samankan S., Çiçek Ö.Y., Altınkaynak A. (2022). Numerical and experimental analysis of optimal nozzle dimensions for FDM printers. Prog. Addit. Manuf..

[118] Chung M., Radacsi N., Robert C., McCarthy E.D., Callanan A., Conlisk N., Hoskins P.R., Koutsos V. (2018). On the optimization of low-cost FDM 3D printers for accurate replication of patient-specific abdominal aortic aneurysm geometry, 3D Print. Med.

[119] Equbal A., Sood A.K., Ansari A.R., Equbal M.A. (2017). Optimization of process parameters of FDM part for minimiizing its dimensional inaccuracy. Int. J. Mech. Prod. Eng. Res. Dev..

[120] Equbal A., Equbal M.I., Sood A.K. (2019). PCA-based desirability method for dimensional improvement of part extruded by fused deposition modelling technology. Prog. Addit. Manuf..

[121] Chang D.-Y., Huang B.-H. (2011). Studies on profile error and extruding aperture for the RP parts using the fused deposition modeling process. Int. J. Adv. Manuf. Technol..

[122] Pérez M., Medina-Sánchez G., García-Collado A., Gupta M., Carou D. (2018). Surface quality enhancement of fused deposition modeling (FDM) printed samples based on the selection of critical printing parameters. Materials.

[123] Aslani K.-E., Chaidas D., Kechagias J., Kyratsis P., Salonitis K. (2020). Quality performance evaluation of thin walled PLA 3D printed parts using the taguchi method and grey relational analysis. J. Manuf. Mater. Process..

[124] Bedi P., Singh R., Ahuja I. (2020). Multifactor optimization of FDM process parameters for development of rapid tooling using SiC/Al 2 O 3 -reinforced LDPE filament. J. Thermoplast. Compos. Mater..

[125] Wolszczak P., Lygas K., Paszko M., Wach R.A. (2018). Heat distribution in material during fused deposition modelling. Rapid Prototyp. J..

[126] Pourali M., Peterson A.M. (2021). Fused filament fabrication of void-free parts using low viscosity hot melt adhesives. Addit. Manuf..

[127] Olam M., Tosun N. (2022). Assessment of 3D printings produced in fused deposition modeling printer using polylactic acid/TiO2/hydroxyapatite composite filaments. J. Mater. Eng. Perform..

[128] Tootooni M.S., Dsouza A., Donovan R., Rao P.K., Kong Z., Borgesen P. (2017). Addit. Manuf. Mater., American Society of Mechanical Engineers (ASME).

[129] Garg A., Bhattacharya A., Batish A. (2016). On surface finish and dimensional accuracy of FDM parts after cold vapor treatment. Mater. Manuf. Process..

[130] Tiwari K., Kumar S. (2018). Analysis of the factors affecting the dimensional accuracy of 3D printed products. Mater. Today Proc..

[131] Latiff Z.A., Rahman M.R.A., Saad F. (2013). Dimensional accuracy evaluation of rapid prototyping fused deposition modeling process of FDM200mc machine on basic engineering profiles. Appl. Mech. Mater..

[132] Singh R. (2013). Some investigations for small-sized product fabrication with FDM for plastic components. Rapid Prototyp. J..

[133] Eswaran P., Sivakumar K., Subramaniyan M. (2018). Minimizing error on circularity of FDM manufactured part. Mater. Today Proc..

[134] Kalyan K., Singh J., Phull G.S., Soni S., Singh H., Kaur G. (2018). Integration of FDM and vapor smoothing process: analyzing properties of fabricated ABS replicas. Mater. Today Proc..

[135] Abdelrhman A.M., Wei Gan W., Kurniawan D. (2019). Effect of part orientation on dimensional accuracy, part strength, and surface quality of three dimensional printed part. IOP Conf. Ser. Mater. Sci. Eng..

[136] Pennington R.C., Hoekstra N.L., Newcomer J.L. (2005). Significant factors in the dimensional accuracy of fused deposition modelling. Proc. Inst. Mech. Eng. Part E J. Process Mech. Eng..

[137] Vyavahare S., Kumar S., Panghal D. (2020). Experimental study of surface roughness, dimensional accuracy and time of fabrication of parts produced by fused deposition modelling. Rapid Prototyp. J..

[138] Lanzotti A., Martorelli M., Staiano G. (2015). Understanding process parameter effects of RepRap open-source three-dimensional printers through a design of experiments approach. J. Manuf. Sci. Eng..

[139] Ippolito R., Iuliano L., Gatto A. (1995). Benchmarking of rapid prototyping techniques in terms of dimensional accuracy and surface finish. CIRP Ann.

[140] Zuza M. (2018). https://blog.prusaprinters.org/everything-about-nozzles-with-a-different-diameter_8344/.

[141] Kondo H. (2020). https://all3dp.com/2/3d-printing-speed-optimal-settings/.

[142] Paul R., Anand S. (2011). Optimal part orientation in Rapid Manufacturing process for achieving geometric tolerances. J. Manuf. Syst..

[143] Feuerbach T., Kock S., Thommes M. (2020). Slicing parameter optimization for 3D printing of biodegradable drug-eluting tracheal stents. Pharmaceut. Dev. Technol..

[144] Messimer S.L., Pereira T.R., Patterson A.E., Lubna M., Drozda F.O. (2019). Full-density fused deposition modeling dimensional error as a function of raster angle and build orientation: large dataset for eleven materials. J. Manuf. Mater. Process..

[145] Hazrat Ali M., Abilgaziyev A. (2021). Fused deposition modeling based 3D printing: design. Ideas, Simulations.

[146] Mohamed O.A., Masood S.H., Bhowmik J.L. (2015). Optimization of fused deposition modeling process parameters: a review of current research and future prospects. Adv. Manuf..

[147] Jaisingh Sheoran A., Kumar H. (2020). Fused Deposition modeling process parameters optimization and effect on mechanical properties and part quality: review and reflection on present research. Mater. Today Proc..

[148] Spoerk M., Holzer C., Gonzalez-Gutierrez J. (2020). Material extrusion-based additive manufacturing of polypropylene: a review on how to improve dimensional inaccuracy and warpage. J. Appl. Polym. Sci..

[149] Comminal R., Serdeczny M.P., Pedersen D.B., Spangenberg J. (2018). Numerical modeling of the strand deposition flow in extrusion-based additive manufacturing. Addit. Manuf..

[150] Comminal R., Serdeczny M.P., Pedersen D.B., Spangenberg J. (2018). Numerical simulation of extrusion-based additive manufacturing–effect of the nozzle geometry on the strand cross-section. Eur. Soc. Precis. Eng. Nanotechnology, Conf. Proc.–18th Int. Conf. Exhib. EUSPEN.

[151] Serdeczny M.P., Comminal R., Pedersen D.B., Spangenberg J. (2018). Experimental validation of a numerical model for the strand shape in material extrusion additive manufacturing. Addit. Manuf..

[152] Hebda M., McIlroy C., Whiteside B., Caton-Rose F., Coates P. (2019). A method for predicting geometric characteristics of polymer deposition during fused-filament-fabrication. Addit. Manuf..

[153] Gleadall A., Ashcroft I., Segal J. (2018). VOLCO: A predictive model for 3D printed microarchitecture. Addit. Manuf..

[154] Golab M., Massey S., Moultrie J. (2021). Ind. Addit. Manuf..

[155] Serdeczny M.P., Comminal R., Pedersen D.B., Spangenberg J. (2019). Numerical simulations of the mesostructure formation in material extrusion additive manufacturing. Addit. Manuf..

[156] Peterson A.M. (2019). Review of acrylonitrile butadiene styrene in fused filament fabrication: a plastics engineering-focused perspective. Addit. Manuf..

[157] Ahn D.K., Kim H.C., Lee S.H. (2005). Determination of fabrication direction to minimize post-machining in FDM by prediction of non-linear roughness characteristics. J. Mech. Sci. Technol..

[158] Saqib S., Urbanic J., ElMaraghy H.A. (2012). Enabling Manuf. Compet. Econ. Sustain..

[159] Zhang J.W., Peng A.H. (2012). Process-parameter optimization for fused deposition modeling based on taguchi method. Adv. Mater. Res..

[160] Kumar Y.R. (2012). An application of Taguchi’s technique to improve the accuracy of rapid prototyped FDM parts. Int. J. Mater. Eng. Innovat..

[161] Górski F., Kuczko W., Wichniarek R. (2013). Influence of process parameters on dimensional accuracy of parts manufactured using fused deposition modelling technology. Adv. Sci. Technol. – Res. J..

[162] Cunico M.W.M. (2013). Study and optimisation of FDM process parameters for support-material-free deposition of filaments and increased layer adherence. Virtual Phys. Prototyp..

[163] Rahmati S., Vahabli E. (2015). Evaluation of analytical modeling for improvement of surface roughness of FDM test part using measurement results. Int. J. Adv. Manuf. Technol..

[164] Fuhrmann M., Falk B., Schmitt R. (2016). Model-based parameter optimization of a fused deposition modelling process. 2016 IEEE Int. Symp. Intell. Control, IEEE.

[165] Kung C., Lee T.-H. (2016). 2016 Int. Symp. Comput. Consum. Control, IEEE.

[166] Vahabli E., Rahmati S. (2017). Improvement of FDM parts’ surface quality using optimized neural networks – medical case studies. Rapid Prototyp. J..

[167] Simsek S., Yaman U. (2016). Dimensional accuracy improvement of fused filament fabrication holes utilizing modified interior. 19th Int. Conf. Electr. Mach. Syst. ICEMS.

[168] Zarko J., Vladic G., Pal M., Dedijer S. (2017). Influence of printing speed on production of embossing tools using FDM 3d printing technology. J. Graph. Eng. Des..

[169] Armillotta A., Bellotti M., Cavallaro M. (2018). Warpage of FDM parts: experimental tests and analytic model. Robot. Comput. Integrated Manuf..

[170] Khan M.S., Dash J.P. (2019). 3D Print. Addit. Manuf. Technol..

[171] Gordeev E.G., Galushko A.S., Ananikov V.P. (2018). Improvement of quality of 3D printed objects by elimination of microscopic structural defects in fused deposition modeling. PLoS One.

[172] Bhowmik S., Jagadish K., Gupta (2019). SpringerBriefs Appl. Sci. Technol..

[173] Alhijjaj M., Nasereddin J., Belton P., Qi S. (2019). Impact of processing parameters on the quality of pharmaceutical solid dosage forms produced by fused deposition modeling (FDM). Pharmaceutics.

[174] Saad M.S., Nor A.M., Baharudin M.E., Zakaria M.Z., Aiman A. (2019). Optimization of surface roughness in FDM 3D printer using response surface methodology, particle swarm optimization, and symbiotic organism search algorithms. Int. J. Adv. Manuf. Technol..

[175] Kasim M.S., Harun N.H., Hafiz M.S.A., Mohamed S.B., Mohamad W.N.F.W. (2019). Multi-response optimization of process parameter in fused deposition modelling by response surface methodology. Int. J. Recent Technol. Eng..

[176] Wilza R., Iskandar, Seprianto D., Adesta E.Y.T. (2019). Optimization of parameters in three-dimensional printing objects with fused deposition modeling technology against geometry accuracy. Int. J. Recent Technol. Eng..

[177] Kuo C.-C., Wu Y.-R., Li M.-H., Wu H.-W. (2019). Minimizing warpage of ABS prototypes built with low-cost fused deposition modeling machine using developed closed-chamber and optimal process parameters. Int. J. Adv. Manuf. Technol..

[178] Yang L., Li S., Li Y., Yang M., Yuan Q. (2019). Experimental investigations for optimizing the extrusion parameters on FDM PLA printed parts. J. Mater. Eng. Perform..

[179] Hriţuc A., Slătineanu L., Mihalache A., Dodun O., Coteaţă M., Nagîţ G. (2020). Accuracy of polylactide parts made by 3D printing. Macromol. Symp..

